# CSF biomarkers of neuroinflammation in distinct forms and subtypes of neurodegenerative dementia

**DOI:** 10.1186/s13195-019-0562-4

**Published:** 2019-12-31

**Authors:** Samir Abu-Rumeileh, Petra Steinacker, Barbara Polischi, Angela Mammana, Anna Bartoletti-Stella, Patrick Oeckl, Simone Baiardi, Corrado Zenesini, André Huss, Pietro Cortelli, Sabina Capellari, Markus Otto, Piero Parchi

**Affiliations:** 1 Department of Biomedical and NeuroMotor Sciences (DIBINEM), University of Bologna, 40139 Bologna, Italy; 2 Department of Neurology, Ulm University Hospital, 89073 Ulm, Germany; 3 Ospedale Bellaria, IRCCS Istituto delle Scienze Neurologiche di Bologna, 40139 Bologna, Italy; 4 Department of Experimental Diagnostic and Specialty Medicine (DIMES), University of Bologna, 40138 Bologna, Italy

**Keywords:** Creutzfeldt-Jakob disease, Alzheimer’s disease, Amyotrophic lateral sclerosis, Corticobasal syndrome, Frontotemporal dementia, Neurofilament light, Progressive supranuclear palsy, Tau protein, Amyloid-beta, Human prion disease

## Abstract

**Background:**

In neurodegenerative dementias (NDs) such as prion disease, Alzheimer’s disease (AD), and frontotemporal lobar degeneration (FTLD), protein misfolding leads to the tissue deposition of protein aggregates which, in turn, trigger neuroinflammation and neurodegeneration. Cerebrospinal fluid (CSF) biomarkers have the potential to reflect different aspects of these phenomena across distinct clinicopathological subtypes and disease stages.

**Methods:**

We investigated CSF glial markers, namely chitotriosidase 1 (CHIT1), chitinase-3-like protein 1 (YKL-40) and glial fibrillary acidic protein (GFAP) in prion disease subtypes (*n* = 101), AD (*n* = 40), clinicopathological subgroups of FTLD (*n* = 72), and controls (*n* = 40) using validated, commercially available ELISA assays. We explored glial biomarker levels’ associations with disease variables and neurodegenerative CSF biomarkers and evaluated their diagnostic accuracy. The genotype of the *CHIT1* rs3831317 polymorphic site was also analyzed.

**Results:**

Each ND group showed increased levels of CHIT1, YKL-40, and GFAP compared to controls with a difference between prion disease and AD or FTLD limited to YKL-40, which showed higher values in the former group. CHIT1 levels were reduced in both heterozygotes and homozygotes for the *CHIT1* 24-bp duplication (rs3831317) in FTLD and controls, but this effect was less significant in AD and prion disease. After stratification according to molecular subgroups, we demonstrated (i) an upregulation of all glial markers in Creutzfeldt-Jakob disease VV2 compared to other disease subtypes, (ii) a difference in CHIT1 levels between FTLD with TAU and TDP43 pathology, and (iii) a marked increase of YKL-40 in FTLD with amyotrophic lateral sclerosis (ALS) in comparison with FTLD without ALS. In prion disease, glial markers correlated with disease stage and were already elevated in one pre-symptomatic case of Gerstmann-Sträussler-Scheinker disease. Regarding the diagnostic value, YKL-40 was the only glial marker that showed a moderate accuracy in the distinction between controls and NDs.

**Conclusions:**

NDs share a CSF profile characterized by increased levels of CSF CHIT1, YKL-40, and GFAP, which likely reflects a common neuroinflammatory response to protein misfolding and aggregation. CSF glial markers of neuroinflammation demonstrate limited diagnostic value but have some potential for monitoring the clinical and, possibly, preclinical phases of NDs.

## Background

Prion disease, frontotemporal lobar degeneration (FTLD), and Alzheimer’s disease (AD) are prototypical neurodegenerative dementias (NDs) characterized by protein misfolding and seeded aggregation. Prion disease, the most heterogeneous and clinically severe of these disorders, encompasses four major clinical-pathological phenotypes, namely, Creutzfeldt-Jakob disease (CJD), Gerstmann-Sträussler-Scheinker disease (GSS), fatal familial insomnia (FFI), and variably protease-sensitive prionopathy (VPSPr), each in turn including a variable number of disease subtypes [1]. CJD, the most common form, includes six major clinicopathological subtypes that are mainly determined by the genotype at the methionine (M)/valine (V) polymorphic codon 129 of the *PRNP* gene and the type (1 or 2) of disease-associated prion protein (PrP^Sc^) accumulating in the brain and named accordingly as MM(V)1, MM2 cortical (MM2C), MM2 thalamic (MM2T), MV2 kuru (MV2K), VV1, and VV2 subtypes [1]. Similarly, FTLD comprises a broad spectrum of clinical syndromes, including the behavioral variant of frontotemporal dementia (bvFTD), primary progressive aphasia (PPA), amyotrophic lateral sclerosis associated with FTD (ALS-FTD), progressive supranuclear palsy (PSP), and corticobasal syndrome (CBS) [2, 3]. Moreover, the heterogeneity of FTLD extends to the underlying molecular pathology, which allows the classification of this disorder into two major subgroups [i.e., FTLD with TDP43 pathology (FTLD-TDP) and FTLD with tau pathology (FTLD-TAU)] [2, 3]. Finally, AD is uniquely characterized by two types of misfolding events which involve proteins amyloid-β (Aβ) and tau forming, respectively, extracellular amyloid plaques and intracellular neurofibrillary degeneration [4]. At variance with prion disease and FTLD, no definite disease subtypes of AD are currently recognized, although clinical variants with an atypical onset and, possibly, pathological variants differing in the molecular properties of Aβ conformers are increasingly reported [4, 5].

Growing evidence indicates that the activation of the innate immune system (also referred to as “neuroinflammation”) is an early pathogenic event across the spectrum of neurodegenerative diseases, including prion disease, AD, and FTLD [6–11]. Activated microglia and astrocytes produce several signaling molecules, such as cytokines, chemokines, and other inflammatory proteins as a reaction to the ongoing deposition of misfolded proteins [8–10, 12]. The results of several studies suggested that the assessment of these proteins in the cerebrospinal fluid (CSF) as surrogate biomarkers of neuroinflammation may contribute knowledge regarding the timing, type, and extent of immune response that occur in NDs. In terms of biomarker value, the most promising results came from studies on chitinase-3-like protein 1 (YKL-40), glial fibrillary acidic protein (GFAP), and chitotriosidase 1 (CHIT1) [13–24]. While YKL-40 and GFAP are well-known markers of astrogliosis, being upregulated in reactive astrocytes [13–21, 23, 24], CHIT1 is a microglia/macrophage protein that cleaves *N*-acetyl glucosamine polymers (mainly found in chitin) in AD amyloid plaques [25] and is highly expressed in ALS spinal cord in association with microglial activation [22, 23]. Of notice, the expression and activity of CHIT1 may be reduced in subjects carrying a polymorphic 24-bp duplication in exon 10 of the CHIT1 gene (rs3831317 polymorphism) [23, 26], which has a high prevalence in European populations (35–50%) [27]. Despite this acquired knowledge, the distribution of values of these biomarkers across different NDs, disease subtypes, and stages of disease progression is not fully understood. Moreover, given the recent development of disease-modifying therapies, such as humanized antibodies which target misfolded proteins and interact with the immune response [10], there is an urgent need for further investigations regarding markers that may be used to monitor the effects of these drugs on the neuroinflammatory response.

In this study, we measured the CSF levels of the glial markers CHIT1, YKL-40 and GFAP and several other biomarkers of neurodegeneration, in AD, prion disease subtypes, and clinicopathological subgroups of FTD/FTLD.

## Materials and methods

### Inclusion criteria and case classification

We retrospectively analyzed 253 CSF samples submitted to the Neuropathology Laboratory at the Institute of Neurological Sciences of Bologna (*n* = 221) or to the Department of Neurology at Ulm University Hospital (*n* = 32) between 2010 and 2018. The cohort comprised 40 healthy controls, 101 patients with prion disease, 40 with AD, and 72 with FTD/FTLD. The 32 samples from Ulm included 1 prion disease and 31 FTD cases. The study was conducted according to the revised Declaration of Helsinki and Good Clinical Practice guidelines. Informed consent was given by study participants or the next of kin. The present study was approved by the ethics committees of “Area Vasta Emilia Centro” (approval number AVEC:18025, 113/2018/OSS/AUSLBO) and Ulm University (approval number 20/10).

For each patient, we collected the clinical history and the results of neurological examination/s (including the evaluation of cognitive status) and of neuroimaging investigations, such as brain computed tomography (CT), brain magnetic resonance imaging (MRI), fluorodeoxyglucose positron emission tomography, and cerebral blood flow single-photon emission computed tomography. For AD and FTD groups, data of Mini-Mental State Examination (MMSE) were also obtained. All data were collected between 2010 and 2019 (June).

Classification of prion diseases was made according to the newly proposed criteria for CJD and related disorders (http://www.cjd.ed.ac.uk/sites/default/files/criteria_0.pdf). Specifically, the group of “definite” prion disease consisted of 65 sporadic cases examined neuropathologically [64 sporadic Creutzfeldt-Jakob disease (sCJD) and 1 VPSPr] and 14 genetic cases carrying a pathogenic *PRNP* mutation [5 genetic CJD (gCJD) E200K, 5 gCJD V210I, 3 FFI (D178N), 2 GSS (P102L) subjects], whereas the group of “probable” sCJD, included 21 patients fulfilling the clinical criteria for possible sCJD and showing either a positive prion RT-QuIC assay or a positive diffusion-weighted/fluid-attenuated inversion recovery (DW/FLAIR)-MRI scan or both (Additional file 1: Table S1). Molecular analysis of the *PRNP* gene, PrP^Sc^ typing, and CJD histotype classification was performed in all autopsied cases according to the established methodologies and consensus criteria [28–30].

For the analysis based on the molecular subtypes, we merged the subjects with definite sCJD MM(V)1 (*n* = 34), VV2 (*n* = 18), MV2K (*n* = 9), MM2C (*n* = 2), and VV1(*n* = 1) diagnosis [31] with those with a probable CJD diagnosis and a high level of certainty of the relative subtype (8 probable VV2, 11 probable MV2K, and 2 probable MM2C). The ultimate classification of probable cases resulted from the consensus of 2 consultant neurologists (SAR and PP), while blinded to the results of CSF biomarkers, after reviewing the typical clinical features, disease duration at death or last follow-up, and the result of codon 129 genotype (MM, MV, and VV) and brain MRI [29–31]. Specifically, a disease duration longer than 6 months in MV or MM cases suggested a diagnosis of probable MV2K or MM2C, respectively (Additional file 1: Table S1).

Additionally, for one GSS case only [32], we examined a CSF sample collected during the pre-symptomatic disease stage at the age of 50 in addition to the one obtained at onset (52 years old).

AD patients were diagnosed according to the International Working Group 2 criteria, including the presence of a characteristic AD CSF biomarker profile, calculated using in-house cutoff values [phosphorylated (p)-tau/Aβ42 ratio > 0.108 and total (t)-tau/Aβ42 ratio > 0.615] [5, 33] (Additional file 1: Table S2). In particular, 35 AD cases fulfilled the criteria for typical AD, 3 for atypical AD-logopenic variant, and 2 for atypical AD posterior variant. Moreover, in 1 autopsied case, the neuropathological assessment revealed an intermediate degree of AD pathology [34]. In AD cases, significant vascular ischemic lesions were excluded based on neuroimaging findings.

The FTD group comprised cases with a pathological and/or genetic diagnosis of FTLD-TDP (*n* = 34) and FTLD-TAU (*n* = 6) and patients with a high level of certainty in their diagnosis and sufficient evidence predicting the underlying TAU pathology (*n* = 32) as previously described [33]. Specifically, the FTLD-TDP group (*n* = 34) included patients with (1) a pathological diagnosis of TDP43 pathology (*n* = 2) and (2) a pathogenic mutation in chromosome 9 open reading frame 72 gene (*C9orf72*) (*n* = 19, including one with pathological diagnosis), progranulin gene (*GRN)* (*n* = 12), or TAR DNA-binding protein 43 gene (*TARDBP*) (*n* = 2). At variance, the FTLD-TAU group (*n* = 20) comprised patients with (1) a pathological diagnosis of tauopathy [PSP, *n* = 1; corticobasal degeneration (CBD), *n* = 1], (2) a pathogenic mutation in microtubule-associated protein tau gene (*MAPT*) (*n* = 4), or (3) a clinical diagnosis of sporadic CBS or PSP (*n* = 32). FTD patients were also classified according to the established clinical criteria in bvFTD (*n* = 17), PPA (*n* = 8), ALS-FTD (*n* = 9), CBS (*n* = 12), and PSP (*n* = 23) [35–39]. Three patients, who met the criteria for bvFTD and/or PPA but also showed extrapyramidal signs (in the presence of a mixed phenotype or not fully satisfying the criteria for CBS or PSP diagnosis), were classified as FTD + parkinsonism [33]. In all FTLD cases, the in vivo evidence of AD pathology was gathered using the AD core CSF biomarkers and in-house calculated cutoff ratios. Specifically, a p-tau/Aβ42 ratio > 0.108 (Bologna) [33] or > 0.08 (Ulm), and a t-tau/Aβ42 ratio > 0.615 (Bologna) [33] or > 0.733 (Ulm) were considered supportive for AD (Additional file 1: Table S2).

The control group included 40 age- and sex-matched subjects lacking any clinical or neuroradiologic evidence of central nervous system disease (e.g., tension-type headache, non-inflammatory polyneuropathies, subjective complaints) and having normal values of p-tau/Aβ42 and t-tau/Aβ42 ratios (Additional file 1: Table S2).

### CSF and genetic analyses

CSF samples were obtained by lumbar puncture (LP) at the L3/L4 or L4/L5 level following a standard procedure, centrifuged in case of blood contamination, divided into aliquots, and stored in polypropylene tubes at − 80 °C until analysis.

CSF CHIT1, YKL-40, GFAP, t-tau, and NfL levels were measured in all cases. For classification purposes, CSF p-tau and Aβ42 analyses were limited to the group of controls, AD, and FTD, while the RT-QuIC to the prion disease group (Additional file 1: Tables S1 and S2). Aβ40 was evaluated in the AD and control cohorts to calculate the ratio of Aβ42 to Aβ40 according to a previously published formula [(Aβ42)/(Aβ40) × 10] [40] (Additional file 1: Table S2). We measured AD core biomarkers prospectively in a routine clinical setting and the neuroinfiammatory markers and NfL in a research setting. Both centers analyzed AD core biomarkers, NfL, CHIT1, and YKL-40 in their own samples, using a comparable pre-analytical protocol and the same enzyme-linked immunosorbent assay (ELISA) kit. Otherwise, the laboratory in Ulm carried out all GFAP assays, and the lab in Bologna, all Aβ40 measurements. Both laboratories participated in the Alzheimer’s Association quality control program on CSF biomarkers [41] and used the same ELISA kits for all analyses. CSF sampling and storage tubes in Bologna were Sarstedt Inc. screw-cup tubes of polypropylene (PP) 10 or 13 ml and Sarstedt screw-cup microtube 0.5 ml PP. The lab in Ulm used the same sampling tubes, while the storage tubes were LVL technologies MX500 screw-cup tubes of PP. To address the inter-laboratory variability in AD core biomarker, we compared the biomarker values in the same diagnostic groups between the Bologna and Ulm cohorts and found no significant differences (see the “Results” section).

YKL-40 was analyzed with the R&D ELISA (R&D Systems, Minneapolis, MN, USA) according to the manufacturer’s instructions. CSF concentrations of CHIT1 and GFAP were measured using ELISA kits (MBL, Belgium; Biovendor, Czech Republic), as previously described [22, 23]. CSF NfL, t-tau, p-tau, Aβ42, Aβ40, and levels were also analyzed using commercially available ELISA kits (IBL, Hamburg, Germany; INNOTEST htau-Ag, INNOTEST phosphorylated-Tau181, INNOTEST Aβ1–42 and INNOTEST Aβ1–40, Innogenetics/Fujirebio Europe, Ghent, Belgium) as previously described [42, 43]. PrP^Sc^ seeding activity was detected by RT-QuIC as previously described [44].

The mean intra- and inter-assay coefficients of variation (CVs) were ≤ 5% and < 20%, respectively, for t-tau, p-tau, Aβ42, Aβ40, and NfL as previously reported [22, 41, 42], and the same was confirmed for CHIT1, YKL-40, and GFAP in both centers.

In a similar cohort, we previously demonstrated that storage time had no effect on CSF NfL, t-tau, p-tau, Aβ42, and Aβ40 and RT-QuIC results [30, 42]. In the present study, we extended these analyses also to CHIT1, YKL-40, and GFAP and found no associations between storage time and the protein levels at univariate linear regression analyses.

Genomic DNA (gDNA) was isolated from the peripheral blood by the Maxwell 16 extractor (Promega, Madison, WI, USA) or from frozen postmortem brain tissue using a standard phenol-chloroform DNA extraction. gDNA was quantified using the Quantus Fluorometer (Promega) with QuantiFluor double-stranded DNA system. We genotyped all cases with available DNA (*n* = 219) to rule out differences in CHIT1 concentrations due to the rs3831317 polymorphism. In detail, the 24-bp duplication of *CHIT1* (c.1049_1072dup, NM_003465.2) was detected by fluorescent polymerase chain reaction using previously reported primers [45]. Amplified fragments were analyzed by capillary electrophoresis (3500Dx Genetic Analyzer, Applied Biosystem), and the peak number and size detected by Gene Mapper Software (Applied Biosystems).

In the present study, ten cases [nine homozygotes (Homo) and one heterozygote (Het) for CHIT1 24-bp duplication] showed unmeasurable CHIT1 levels; these values were approximated to the detection limit of the assay (280 pg/ml). Moreover, to exclude the effect of genotype on CHIT1 levels, we performed the analyses regarding CSF CHIT1 levels twice, in (i) all cases (*n* = 253) and (ii) those with wild-type (WT) and/or heterozygous status for CHIT1 24-bp duplication (*n* = 207).

### Statistical analyses

Statistical analysis was performed using IBM SPSS Statistics version 21 (IBM, Armonk, NY, USA), Stata Stata SE version 14.2 (StataCorp LLC, Texas, USA) and GraphPad Prism 7 (GraphPad Software, La Jolla, CA) software. Based on the presence or not of a normal distribution of the values, data were expressed as mean ± standard deviation (SD) or median and interquartile range (IQR). For continuous variables, depending on the data distribution, the Mann-Whitney *U* test or the *t* test were used to test the differences between the two groups, while the Kruskal-Wallis test (followed by Dunn-Bonferroni post hoc test) or the one-way analysis of variance (ANOVA) (followed by Tukey’s post hoc test) was applied for multiple group comparisons. Chi-square test was adopted for categorical variables. All reported *p* values have been adjusted for multiple comparison analyses. Multivariate linear regression models were used to adjust (for age and sex) the differences in CSF biomarkers between the groups, after the transformation of the dependent variable in the logarithmic scale. Receiver Operating Characteristic (ROC) analyses were performed to establish the diagnostic accuracy, sensitivity, and specificity of each biomarker. The optimal cutoff value for biomarkers was chosen using the maximized Youden index. The Youden index for a cutoff is defined by its sensitivity + specificity − 1. Univariate linear regression models with Pearson’s correlations or Spearman’s correlations were used to test the possible associations between analyzed variables. Differences were considered statistically significant at *p* <  0.05.

## Results

### Demographics of diagnostic groups and effect of demographic variables on CSF biomarkers

Demographic data and *CHIT1* genotypes for each diagnostic group are shown in Table 1. There were no significant differences regarding sex distribution between the groups. The age slightly differed between the diagnostic groups (*p* = 0.049), but post hoc testing only revealed a significant difference before the multiple comparison adjustment, between FTD and AD (*p* = 0.023) or FTD and prion disease (*p* = 0.018). As expected, given the frequent subacute onset and the rapid clinical progression, the time interval between onset and LP was significantly shorter in subjects with prion disease than in those with AD or FTD (*p* < 0.001 for each comparison). The MMSE score showed a significant difference between FTD and AD patients (*p* < 0.001), as previously reported [46].

**Table 1:** Demographic data and CHIT1 genotype in the diagnostic groups

Diagnosis	Prion disease	AD	FTD	Controls	*P*
*N*	101	40	72	40	
Age at LP (years ± SD)	67.44 ± 9.30	68.63 ± 8.16	63.99 ± 9.04	64.88 ± 9.62	0.049^a^
Female (%)	46.5%	40.0%	59.7%	45%	0.230^b^
Time from onset to LP (months ± SD)	4.34 ± 4.01	44.87 ± 30.08	35.69 ± 26.87	**–**	< 0.001^a^
MMSE score (points ± SD)	–	20.22 ± 4.87	26.50 ± 3.01	–	< 0.001^c^
*CHIT1* genotype					
*N* (%)	98	34	64	23	
WT	58 (59.1)	19 (55.9)	44 (68.8)	12 (52.2)	0.785^b^
Het	34 (34.7)	13 (38.2)	17 (26.6)	10 (43.5)	
Homo	6 (6.1)	2 (5.9)	3 (4.7)	1 (4.3)	

*AD* Alzheimer’s disease, *CHIT1* chitotriosidase 1, *CJD* Creutzfeldt-Jakob disease, *FTD* frontotemporal dementia, *GFAP* glial fibrillary acidic protein, *Het* heterozygotes for CHIT1 24-bp duplication, *Homo* homozygotes for CHIT1 24-bp duplication, *IQR* interquartile range, *LP* lumbar puncture, *MMSE* Mini-Mental State Examination, *N* number, *SD* standard deviation, *YKL-40* chitinase-3-like protein 1, *WT* wild type for *CHIT1* 24-bp duplication

^a^Kruskal-Wallis test

^b^Chi-square test

^c^Mann-Whitney *U* test

In each diagnostic category, the frequency of *CHIT1* genotypes fit the Hardy-Weinberg equilibrium (prion disease: *p* = 0.735; AD: *p* = 0.909; FTD: *p* = 0.429; controls: *p* = 0.541). Moreover, there were no differences between the groups in the frequencies of the *CHIT1* 24-bp duplication, no effect of sex on CSF biomarker levels, and no significant correlation between age and biomarker values except for YKL-40 in controls (Spearman’s rho = 0.446, *p* = 0.004) [23].

Owing to the non-normal distribution of biomarker values and the presence of outliers, Mann-Whitney *U* or Kruskal-Wallis test (followed by Dunn-Bonferroni’s post hoc test) were used for multiple comparisons between two or more patient groups, respectively. Age and sex adjustments were applied.

### CSF biomarkers of neuroinflammation and neurodegeneration in the diagnostic groups

**Fig. 1:**
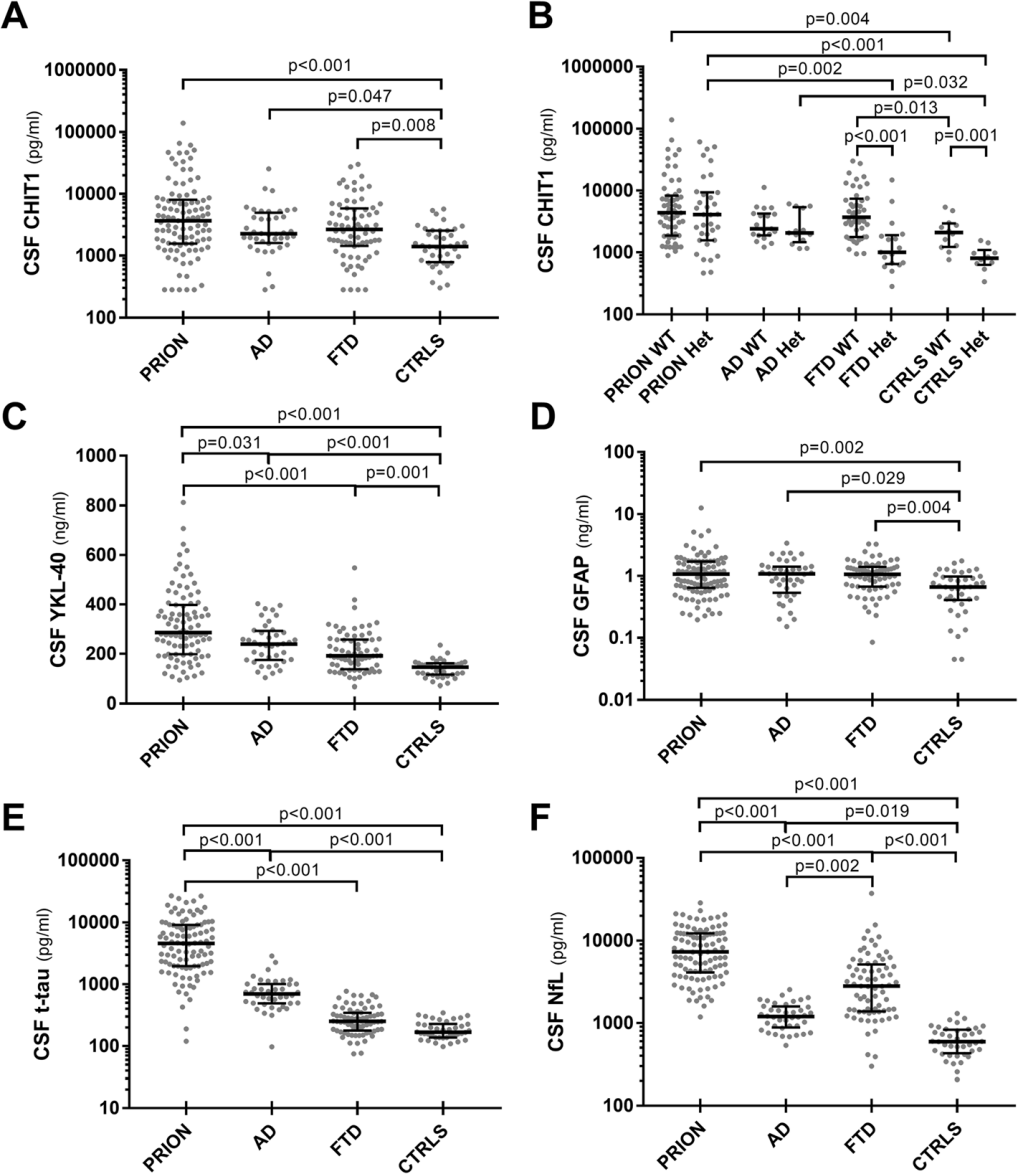
CSF markers of neuroinflammation and neurodegeneration across diagnostic groups. **a** CSF CHIT1, **b** CHIT1 according to genotype (WT: wild type for CHIT1 24-bp duplication; Het: heterozygotes for CHIT1 24-bp duplication), **c** YKL-40, **d** GFAP, **e** t-tau, and **f** NfL levels in prion disease (PRION), Alzheimer’s disease (AD), frontotemporal dementia (FTD), and controls (CTRLS). Horizontal lines represent medians. CHIT1,GFAP, t-tau and NfL values are expressed in logarithmic scale. Only statistically significant differences are displayed (Kruskal-Wallis test followed by Dunn-Bonferroni post hoc test)

The results of the biomarker analyses according to the diagnostic groups are summarized in Table 2 and Fig. 1.

**Table 2:** Biomarkers of neuroinflammation and neurodegeneration in the diagnostic groups

Diagnosis	Prion disease	AD	FTD	Controls	*P*
*N*	101	40	72	40	
CHIT1 all (pg/ml), median (IQR)	3669 (1568–8017)	2259 (1603–4944)	2657 (1437–5800)	1409 (783–2537)	< 0.001^a^
*WT* (pg/ml), median (IQR)	4382 (1874–8311)	2399 (1884–4247)	3706 (1776–7374)	2095 (1226–2948)	0.016^a^
*Het* (pg/ml), median (IQR)	4078 (1570–9379)	2065 (1465–5409)	1006 (648–1894)	803 (628–1090)	< 0.001^a^
YKL-40 (ng/ml), median (IQR)	315 (222–453)	240 (176–293)	192 (135–257)	145 (115–161)	< 0.001^a^
GFAP (ng/ml), median (IQR)	1.028 (0.636–1.698)	1.081 (0.534–1.422)	1.065 (0.667–1.422)	0.665 (0.409–0.978)	0.002^a^
t-tau (pg/ml), median (IQR)	4644 (1977–9223)	698 (491–1013)	253 (179–347)	168 (138–228)	< 0.001^a^
NfL (pg/ml), median (IQR)	7225 (3879–12,188)	1405 (942–1730)	2805 (1382–5158)	595 (430–831)	< 0.001^a^

*AD* Alzheimer’s disease, *CHIT1* chitotriosidase 1, *CJD* Creutzfeldt-Jakob disease, *FTD* frontotemporal dementia, *GFAP* glial fibrillary acidic protein, *Het* heterozygotes for CHIT1 24-bp duplication, *IQR* interquartile range, *N* number, *NfL* neurofilament light chain protein, *t-tau* total tau protein, *YKL-40* chitinase-3-like protein 1, *WT* wild type for CHIT1 24-bp duplication

^a^Kruskal-Wallis test

Each ND cohort showed higher CHIT1 levels than controls (prion disease vs. controls *p* < 0.001, FTD vs. controls *p* = 0.010, AD vs. controls *p* = 0.047) (Table 2, Fig. 1a), although the concentration of the marker did not significantly differ between patients with prion disease, AD, and FTD, findings that were also confirmed after age and sex adjustment (Additional file 1: Table S3). Moreover, the results did not change after excluding the homozygotes for the *CHIT1* 24-bp duplication (prion disease vs. controls *p* < 0.001, AD vs. controls *p* = 0.046, FTD vs. controls *p* = 0.006). CHIT1 levels were reduced in all homozygotes for the 24-bp duplication indipendently from the diagnosis. Otherwise, the heterozygotes for the 24-bp duplication showed significantly reduced levels of CHIT1 in comparison with the non-carriers in both FTD and controls but not in prion disease and AD (Table 2, Fig. 1b).

Patients with NDs had also higher levels of CSF YKL-40 than controls (prion disease vs. controls *p* < 0.001, AD vs. controls *p* < 0.001, FTD vs. controls *p* = 0.001), with subjects with prion disease reaching the highest median levels (prion disease vs. AD *p* = 0.031, prion disease vs. FTD *p* < 0.001), while AD and FTD showed comparable concentrations of the biomarker (Table 2, Fig. 1c); these findings were confirmed after age and sex adjustment (Additional file 1: Table S3). Similarly, all ND groups showed higher levels of GFAP than controls (prion disease vs. controls *p* = 0.002, FTD vs. controls *p* = 0.004, AD vs. controls *p* = 0.029), with no significant differences among the disease groups (Table 2, Fig. 1d), even after age and sex adjustment (Additional file 1: Table S3). Differences in CSF t-tau and NfL among the diagnostic groups are shown in Table 2, Fig. 1e, f, and Additional file 1: Table S3

### CSF levels of glial markers vary across the prion disease phenotypic spectrum (Table 3)

To systematically analyze the biomarker levels across the CJD spectrum, we stratified the sCJD cases according to the molecular subtype [MM(V)1, MV2K, and VV2] [31], the corresponding prion strain [strain M1 = MM(V)1 subtype; strain V2 = VV2 and MV2K subtypes] [1], and the codon 129 genotype (MM, MV, and VV) [31].

**Table 3:** CSF biomarkers of neuroinflammation in prion disease subtypes

Subtype	Number	CHIT1 all (pg/ml), median (IQR)	CHIT1 WT + Het (pg/ml), median (IQR)	YKL-40 (ng/ml), median (IQR)	GFAP (ng/ml), median (IQR)
All sCJD	85	4092 (1509–8644)	4154 (1872–8645)	321 (222–471)	1.115 (0.633–1.655)
*sCJD MM(V)1*	34	3069 (1229–6921)	3290 (1235–7191)	259 (175–358)	0.810 (0.611–1.295)
*sCJD VV2*	26	5060 (2474–15,725)	5718 (2877–30,500)	533 (314–783)	1.638 (0.857–2.559)
*sCJD MV2K*	20	5064 (2170–8532)	5064 (2170–8532)	321 (214–406)	0.763 (0.352–1.230)
*sCJD MM2C*	4	2158 (646–6125)	2572	195 (178–314)	1.146 (0.577–1.716)
*sCJD VV1*	1	38,000	38,000	455	0.829
VPSPr	1	2562	2562	341	2.779
gCJD E200K	5	2572 (828–4415)	2767	269 (206–782)	1.127 (0.955–2.088)
gCJD V210I	5	1820 (934–4415)	1820 (1226–3838)	349 (184–472)	0.412 (0.329–1.351)
FFI (D178N)	3	16,800, 1571, 4156	16,800,1571, 4156	146, 253, 165	0.827, 0.415, 0.227
Pre-symptomatic GSS (P102L)	1	8353*	8353*	186*	1.114*
Symptomatic GSS (P102L)	2	18000*, 2356	18000*, 2356	297*, 450	1.712*, 0.418

*CHIT1* chitotriosidase 1, *CJD* Creutzfeldt-Jakob disease, *FFI* fatal familial insomnia, *gCJD* genetic Creutzfeldt-Jakob disease, *GFAP* glial fibrillary acidic protein, *GSS* Gerstmann-Sträussler-Scheinker syndrome, *Het* heterozygotes for CHIT1 24-bp duplication, *IQR* interquartile range, *LP* lumbar puncture, *MM(V)1* methionine homozygosity (valine) and scrapie prion protein type 1, *MM2C* methionine homozygosity and scrapie prion protein type 2, cortical type *MM2T* methionine homozygosity and scrapie prion protein type 2, thalamic type, *MV2K* methionine/valine heterozygosity and scrapie prion protein type 2, kuru type, *sCJD* sporadic Creutzfeldt-Jakob disease, *VPSPr* variably protease-sensitive prionopathy, *VV1* valine homozygosity and scrapie prion protein type 1, *VV2* valine homozygosity and scrapie prion protein type 2, *WT* wild type for CHIT1 24-bp duplication, *YKL-40* chitinase-3-like protein 1

*The same GSS case

**Fig. 2:**
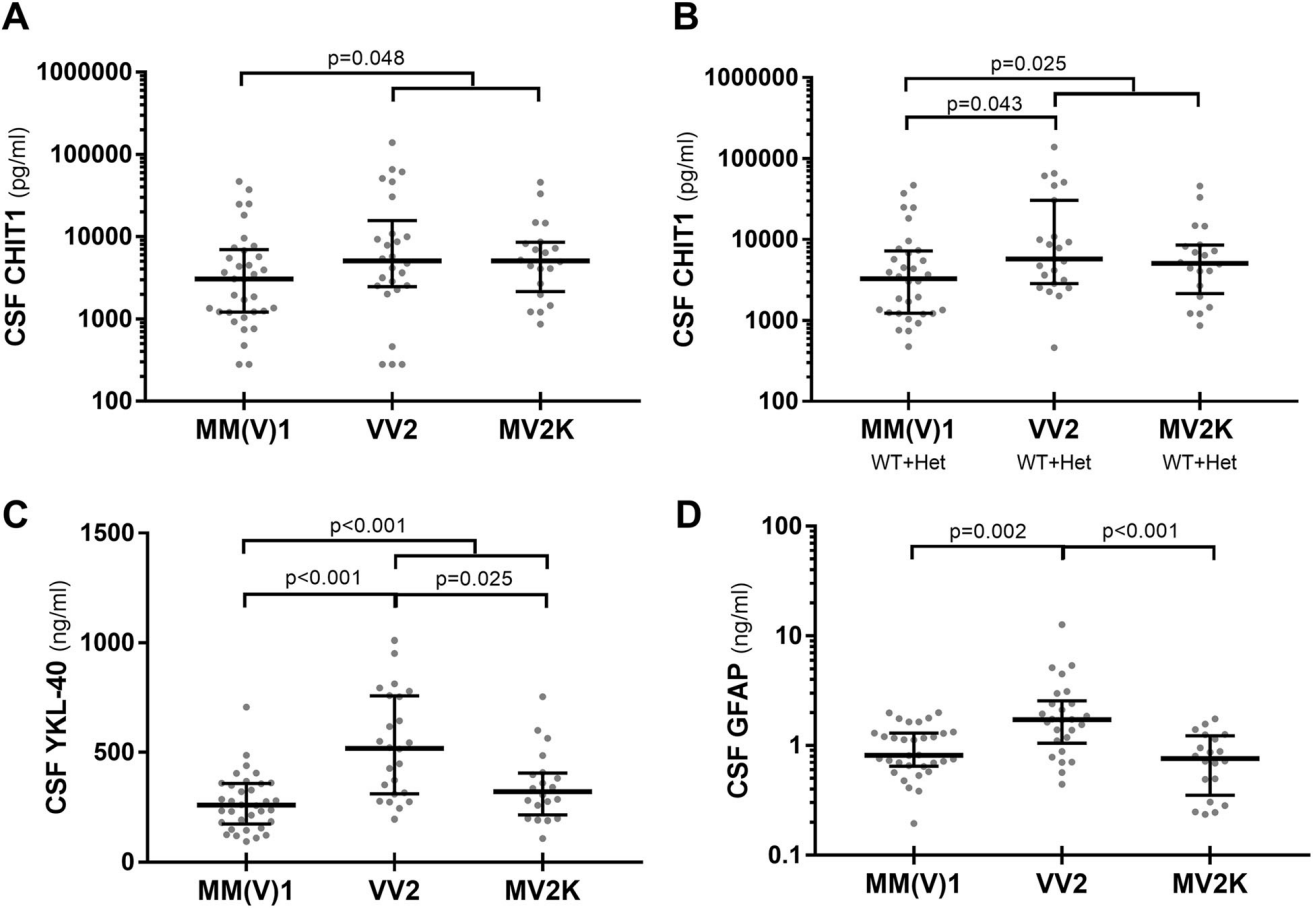
Glial markers in distinct sCJD molecular subtypes. **a** CSF CHIT1 (all cases), **b** CSF CHIT1 (after the exclusion of the homozygotes for CHIT1 24-bp duplication), **c** YKL-40, and **d** GFAP in sCJD MM(V)1, VV2, and MV2K subtypes. Horizontal lines represent medians. CHIT1 and GFAP values are expressed in logarithmic scale. Only statistically significant differences are displayed (Kruskal-Wallis test followed by Dunn-Bonferroni post hoc test)

CJD patients linked to the V2 strain (26 VV2 and 20 MV2K) showed significantly higher CHIT1 levels than those related to the M1 strain [34 MM(V)1] (*p* = 0.048; *p* = 0.025 after the exclusion of the homozygotes for the 24-bp duplication) (Fig. 2a, b). The comparison of CHIT1 levels between CJD subtypes revealed higher values in VV2 subjects than in MM(V)1 cases (*p* = 0.043), but only after the exclusion of the homozygotes for the 24-bp duplication (Fig. 2b).

Regarding YKL-40, sCJD linked to the V2 strain showed increased values compared to those associated with the M1 strain (*p* < 0.001) (Fig. 2c). Again, VV2 subjects demonstrated the highest levels among sCJD subtypes [VV2 vs. MM(V)1 *p* < 0.001, VV2 vs. MV2K *p* = 0.025] (Fig. 2c).

The VV2 group also showed significantly higher GFAP levels in comparison with the MM(V)1 (*p* = 0.002) and MV2K (*p* < 0.001) groups (Fig. 2d), but no difference was detected between strain M1 and V2 (Fig. 2d).

Subanalyses according to the codon 129 genotype are shown in Additional file 1: Figure S1. The comparison between sporadic and genetic prion diseases failed to reveal significant differences in glial marker values. CSF biomarkers of neurodegeneration among prion disease subtypes are shown in Additional file 1: Table S4 and Figure S2. The comparisons regarding glial and neurodegenerative markers among CJD strains and subtypes were confirmed after age and sex adjustment (Additional file 1: Table S5).

Interestingly, the pre-symptomatic GSS case, which was heterozygotes for *CHIT1* 24-bp duplication, showed significantly higher CHIT1 levels than the controls carrying the same *CHIT1* genotype (7.7-fold). At variance, YKL-40 and GFAP only showed slightly higher values (1.1 and 1.2-fold, respectively, compared to controls) (Table 3). After disease onset, CHIT1 levels increased significantly (2.2-fold) while YKL-40 and GFAP elevations were less pronounced (1.6- and 1.5-fold, respectively) (Table 3). Finally, in GSS, NfL showed high levels in the preclinical phase and a further increase after onset (Additional file 1: Table S4).

### CSF biomarkers of neuroinflammation within the FTD/FTLD spectrum (Table 4)

For the FTD/FTLD group, we considered the p-tau/t-tau ratio as a further marker because significantly different levels were described between FTLD-TDP and TAU [33, 47, 48].

**Table 4:** CSF biomarkers of neuroinflammation in the FTD/FTLD spectrum

	Number	CHIT1 all (pg/ml), median (IQR)	CHIT1 WT + Het (pg/ml), median (IQR)	YKL-40 (ng/ml), median (IQR)	GFAP (ng/ml), median (IQR)
Clinical diagnosis					
bvFTD	17	3178 (2008–6437)	3877 (2178–6713)	186 (120–254)	1.365 (0.784–1.977)
PPA	8	2390 (1642–4306)	2390 (1642–4306)	228 (138–268)	1.101 (0.877–1.252)
*nfvPPA*	6	2934 (1657–6597)	2934 (1657–6597)	209 (127–273)	1.054 (0.800–1.373)
*svPPA*	2	1120, 2959	1120, 2959	205, 272	1.016, 1.260
ALS-FTD	9	11,500 (6309–16,048)	14,900 (6671–16,789)	290 (228–392)	0.867 (0.565–1.326)
PSP	23	1767 (929–3074)	1767 (968–3135)	192 (151–242)	0.971 (0.463–1.314)
CBS	12	1659 (825–4391)	1659 (1220–5471)	161 (135–192)	1.200 (0.687–1.413)
FTD + parkinsonism	3	1744, 941, 19,300	1744, 941, 19,300	176, 154, 134	1.577, 0.672, 1.248
FTLD proteinopathies					
FTLD-TAU	38	1766 (987–3237)	1732 (1092–3237)	186 (148–225)	1.002 (0.649–1.324)
FTLD-TDP	34	4484 (1930–11,625)	4575 (1966–12,000)	209 (134–264)	1.226 (0.676–1.686)
*TDP without ALS*	25	3120 (1743–6437)	3149 (1703–6713)	180 (132–254)	1.248 (0.784–1.792)
* TDP with ALS*	9	11,500 (6309–16,048)	14,900 (6671–16,789)	290 (228–392)	0.867 (0.565–1.326)

*ALS* amyotrophic lateral sclerosis, *ALS-FTD* amyotrophic lateral sclerosis associated with frontotemporal dementia, *bvFTD* behavioral variant of frontotemporal dementia, *CBS* corticobasal syndrome, *CHIT1* chitotriosidase 1, *FTD* frontotemporal dementia, *FTLD-TAU* frontotemporal lobar degeneration with tau pathology, *FTLD-TDP* frontotemporal lobar degeneration with TDP43 pathology, *GFAP* glial fibrillary acidic protein, *Het* heterozygotes for CHIT1 24-bp duplication, *IQR* interquartile range, *nfvPPA* nonfluent/agrammatic variant of primary progressive aphasia, *PPA* primary progressive aphasia, *PSP* progressive supranuclear palsy, *svPPA* semantic variant of primary progressive aphasia, *WT* wild type for CHIT1 24-bp duplication, *YKL-40* chitinase-3-like protein 1

**Fig. 3:**
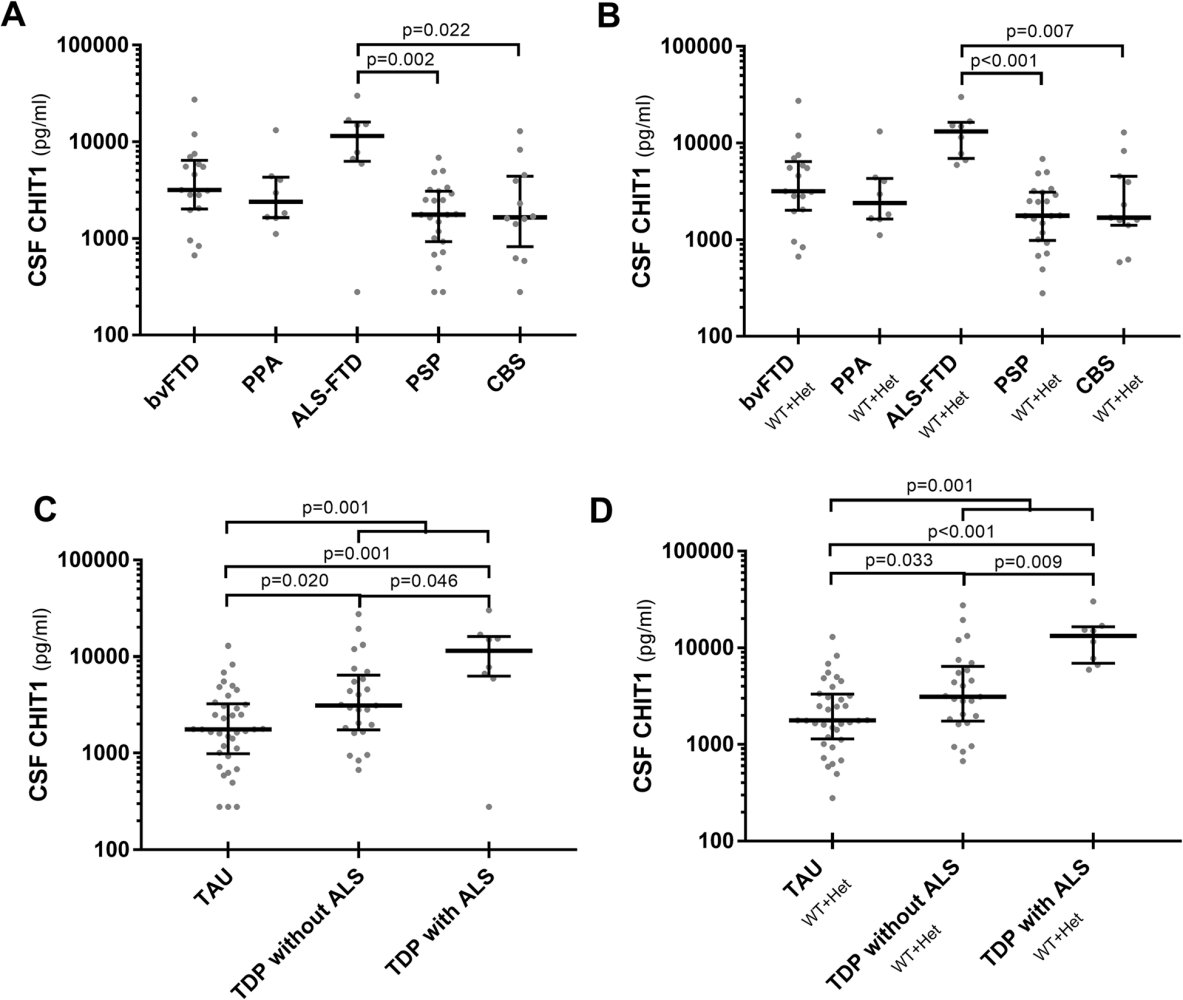
CHIT1 in distinct FTD clinical syndromes and molecular subtypes. **a** CSF CHIT1 (all cases) in FTD clinical groups. **b** CSF CHIT1 (after the exclusion of the homozygotes for CHIT1 24-bp duplication) in FTD clinical groups. **c** CSF CHIT1 in FTLD-TAU, FTLD-TDP without ALS, and FTLD-TDP with ALS (all cases). **d** CSF CHIT1 in FTLD-TAU, FTLD-TDP without ALS, and FTLD-TDP with ALS (after the exclusion of the homozygotes for CHIT1 24-bp duplication). Horizontal lines represent medians. CHIT1 values are expressed in logarithmic scale. Only statistically significant differences are displayed (Kruskal-Wallis test followed by Dunn-Bonferroni post hoc test)

Among the FTD clinical syndromes, CSF CHIT1 levels showed higher values in ALS-FTD than in CBS (*p* = 0.022) or PSP (*p* = 0.002) (Fig. 3a, b). Interestingly, FTLD-TDP subjects showed higher CHIT1 levels than those with FTLD-TAU (*p* = 0.001) (Fig. 3c). The analysis demonstrated increased CHIT1 levels in comparison with TAU not only in TDP with ALS but also in TDP without ALS (*p* = 0.001 and *p* = 0.020, respectively), with the former showing higher values than the latter (*p* = 0.046) (Fig. 3c). The similar genotype distribution between TDP and TAU groups ruled out any effect of the *CHIT1* 24-bp duplication on these results (chi-square) (Additional file 1: Table S6); indeed, we also confirmed the same findings in the group of non-carriers and/or heterozygotes (Fig. 3d, Additional file 1: Table S6). In detail, after excluding the homozygotes for the CHIT1 24-bp duplication, higher CHIT1 levels in comparison with TAU were detected not only in TDP with ALS but also in TDP without ALS (*p* < 0.001 and *p* = 0.033, respectively), with the former showing higher values than the latter (*p* = 0.009) (Fig. 3d). However, when the comparison was limited to definite TAU cases (one definite PSP, one definite CBD, and four *MAPT* carriers), we detected only a tendency towards different CHIT1 values (*p* = 0.086) between TDP and TAU (wild type and heterozygotes).

**Fig. 4:**
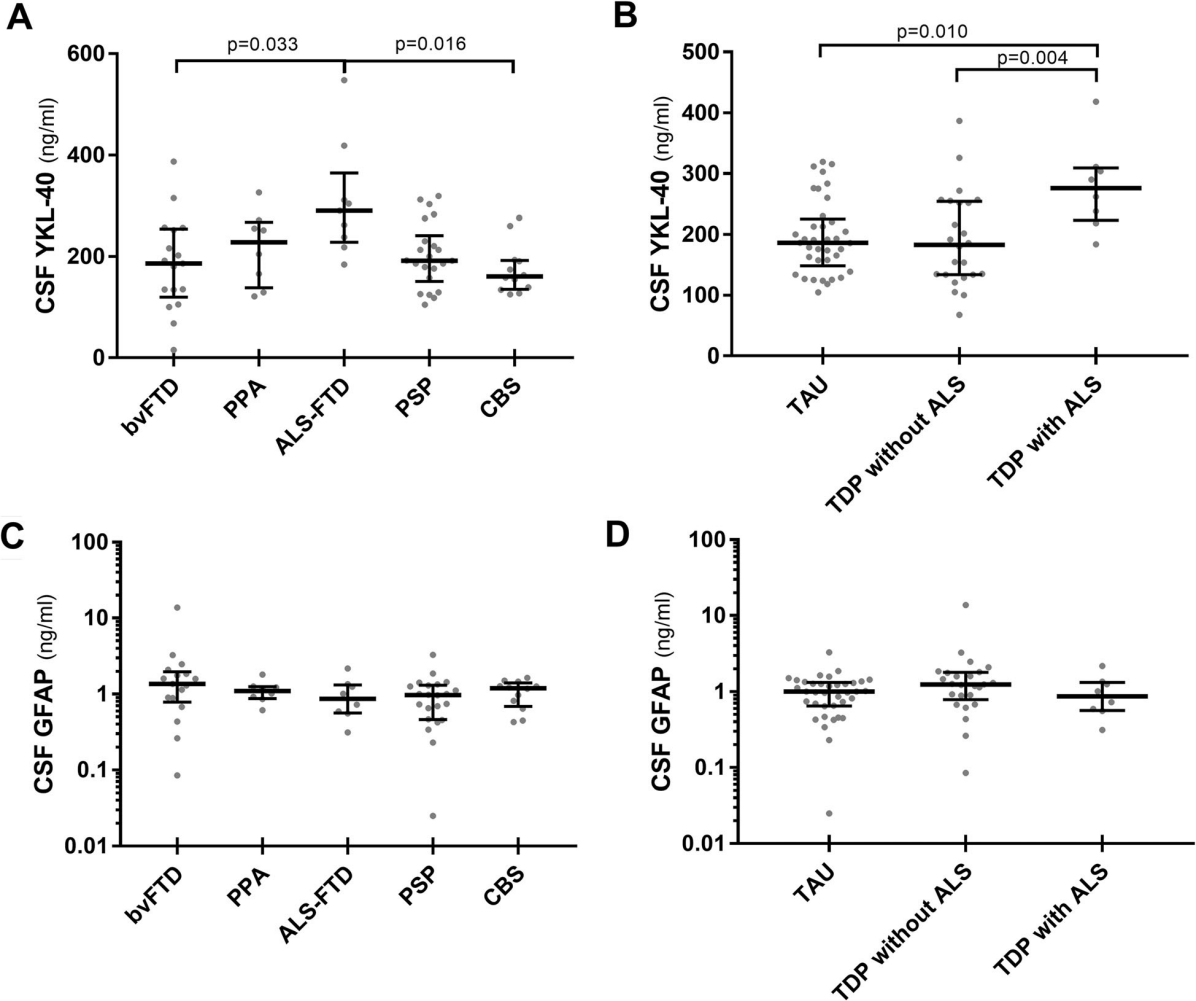
YKL-40 and GFAP in distinct FTD clinical syndromes and molecular subtypes. **a** CSF YKL-40 in FTD clinical groups. **b** CSF YKL-40 in FTLD-TAU, FTLD-TDP without ALS, and FTLD-TDP with ALS. **c** CSF GFAP in FTD clinical groups. **d** CSF GFAP in FTLD-TAU, FTLD-TDP without ALS, and FTLD-TDP with ALS. Horizontal lines represent medians. GFAP values are expressed in logarithmic scale. Only statistically significant differences are displayed (Kruskal-Wallis test followed by Dunn-Bonferroni post hoc test)

YKL-40 showed higher values in FTD-ALS than in bvFTD (*p* = 0.033) and CBS (*p* = 0.016) (Fig. 4a), as well as in TDP with ALS in comparison with TDP without ALS (*p* = 0.004) and FTLD-TAU (*p* = 0.010) (Fig. 4b). Finally, GFAP showed comparable values among the FTD clinical syndromes (Fig. 4c) and proteinopathies (Fig. 4d).

The values of the CSF biomarkers of neurodegeneration in the FTD spectrum are included in Additional file 1: Table S7 and the comparisons in Additional file 1: Figure S3. All the comparisons regarding glial and neurodegenerative markers among FTLD molecular subtypes were confirmed after age and sex adjustment (Additional file 1: Table S8), whereas the adjusted comparisons among FTD clinical groups and subclasses of proteinopathies (e.g., ALS-FTD/TDP with ALS) were not performed due to the small sample size of some groups. The values of the CSF biomarkers in the FTD mutation carriers are included in Additional file 1: Table S9.

To address inter-laboratory variability, we compared biomarker values between Bologna and Ulm FTD cohorts after stratification according to the diagnosis of the most numerous shared groups (bvFTD, PSP, FTLD-TDP, and FTLD-TAU) and did not found significant differences. Moreover, after excluding the Ulm cohort, the large majority of our results were still the same, see Additional file 1: Supplementary text for further details.

### Associations between glial markers, disease variables, and neurodegenerative markers

Overall, the disease duration did not correlate with any biomarker value in the prion disease group. However, there were significant correlations between biomarker values and the disease stage. To estimate the latter parameter, we divided the time from onset to LP by the disease duration as previously described [49]. In the prion disease group, the disease stage correlated with CHIT1 (Spearman’s rho = 0.287; *p* = 0.009), YKL-40 (Spearman’s rho = 0.366, *p* = 0.001), and t-tau (Spearman’s rho = 0.354, *p* = 0.001) values. A further analysis limited to the most representative homogenous prion group, namely the sCJD MM(V)1 subtype, confirmed the findings for CHIT1 and YKL-40 but not for t-tau. The time from onset to LP was not associated with biomarker values in the other disease groups.

We also found some significant correlations between CHIT1, YKL-40, GFAP, and the markers of neurodegeneration (NfL, t-tau, p-tau, Aβ42, and Aβ40) in all diagnostic groups (Additional file 1: Supplementary text). Finally, the MMSE score showed a moderate inverse correlation only with YKL-40 (Spearman’s rho = − 0.497, *p* = 0.007) in AD patients, while it was not associated with any biomarker value in the FTD group.

### Diagnostic values of biomarkers of neuroinflammation and neurodegeneration

Detailed results of the ROC analyses for biomarkers of neuroinflammation and neurodegeneration are shown in Table 5. Among the three neuroinflammatory markers, YKL-40 yielded a good diagnostic value in the discrimination between controls and prion disease (AUC 0.919 ± 0.023) or AD (AUC 0.882 ± 0.038), with at least 80% sensitivity and 80% specificity in both comparisons. However, the same analysis showed a low diagnostic value in the distinction between controls and FTD patients (AUC 0.777 ± 0.043). All three glial markers demonstrated lower accuracy than t-tau or NfL in the distinction between prion disease, AD, and FTD, due to the large overlap in the glial marker levels between the three NDs (data not shown). Finally, the diagnostic accuracy of both NfL and p-tau/t-tau in the discrimination between FTLD-TDP and FTLD-TAU (AUC NfL, 0.827 ± 0.053; p-tau/t-tau, 0.818 ± 0.050) exceeded by far that of CHIT1 (AUC 0.727 ± 0.061).

**Table 5:** Diagnostic value of CSF biomarkers in the differential diagnosis between disease groups

	AUC	Cutoff	sens (%)	spec (%)	AUC	Cutoff	sens (%)	spec (%)
	Prion disease vs. controls				AD vs. controls			
CHIT1	0.746 ± 0.041	> 1664 pg/ml	73.3	62.5	0.701 ± 0.059	> 1911 pg/ml	67.5	65.0
YKL-40	0.919 ± 0.023	> 184 ng/ml	84.8	95.0	0.882 ± 0.038	> 165 ng/ml	82.5	80.0
GFAP	0.687 ± 0.046	> 0.782 ng/ml	62.6	62.5	0.681 ± 0.060	> 0.799 ng/ml	67.5	62.5
NfL	0.990 ± 0.010	> 1458 pg/ml	98.0	100.0	0.888 ± 0.035	> 813 pg/ml	80.0	75.0
t-tau	0.987 ± 0.010	> 388 pg/ml	98.0	100.0	0.973 ± 0.025	> 314 pg/ml	97.5	95.0
	FTD vs. controls				FTLD-TDP vs. FTLD-TAU			
CHIT1	0.688 ± 0.050	> 1616 pg/ml	72.9	62.5	0.727 ± 0.061	> 2657 pg/ml	69.7	68.4
YKL-40	0.777 ± 0.043	> 156 ng/ml	70.0	72.5	0.542 ± 0.071	> 192 ng/ml	54.5	56.8
GFAP	0.706 ± 0.050	> 0.797 ng/ml	67.1	62.5	0.584 ± 0.069	> 1.130 ng/ml	57.6	59.5
NfL	0.949 ± 0.022	> 1037 pg/ml	93.0	92.5	0.827 ± 0.053	> 3040 pg/ml	75.8	81.6
t-tau	0.725 ± 0.048	> 209 pg/ml	67.1	67.5	0.731 ± 0.062	> 253 pg/ml	69.7	68.4
p-tau/t-tau	0.760 ± 0.044	< 0.175	67.5	66.3	0.818 ± 0.050	< 0.141	76.3	72.7

*AD* Alzheimer’s disease, *AUC* area under the curve, *CHIT1* chitotriosidase 1, *CJD* Creutzfeldt-Jakob disease, *FTD* frontotemporal dementia, *FTLD-TAU* frontotemporal lobar degeneration with tau pathology, *FTLD-TDP* frontotemporal lobar degeneration with TDP43 pathology, *GFAP* glial fibrillary acidic protein, *NfL* neurofilament light protein, *p-tau* phosphorylated tau protein, *sens* sensitivity, *spec* specificity, *t-tau* total tau protein, *YKL-40* chitinase-3-like protein 1

## Discussion

The results of the present study document a significant increase in CSF levels of CHIT1, YKL-40, and GFAP in three prototypic human brain proteinopathies likely reflecting the shared significant microglial and astrocytic activation and the advanced neurodegeneration that characterizes the symptomatic phase of these disorders. However, some clinical and pathological subtypes of both prion disease and FTD/FTLD showed significantly higher CSF levels of glial markers, which, in turn, correlated with the disease stage. Overall, these glial markers showed some potential in monitoring the clinical and preclinical phases of the disease, but a limited value in the differential diagnosis of these disorders.

Our finding of increased CSF CHIT1 and GFAP levels in prion disease, AD, and FTD compared to controls, but without significant differences between the three NDs, adds consistency to previous studies in smaller cohorts [14, 22–24]. Interestingly, we confirmed that CHIT1 levels are reduced in FTD and controls that are heterozygous or homozygous for the *CHIT1* 24-bp duplication [23] but also found that the CSF levels did not differ between wild-types and *CHIT1* 24-bp duplication heterozygotes in AD and prion disease. Therefore, we speculate that the significant CHIT1 increase that occurs in prion disease and AD might compensate for the “decrease effect” linked to *CHIT1* 24-bp duplication in exon 10. However, in FTD, CHIT1 levels should be considered reliable only if the genotype is also assessed, because the low protein concentration that is often associated with the *CHIT1* 24-bp duplication might be erroneously interpreted as a negative finding.

Furthermore, we confirmed previous evidence of a more pronounced elevation of YKL-40 in prion disease compared to AD and the lack of a difference in YKL-40 levels between FTD and AD [15, 18–21], which is also supported by the presence of comparable degrees of YKL-40 immunoreactivity in the brains with CBD, PSP, and AD [50].

In prion disease, the significant heterogeneity of the rate of disease progression and neuropathological profiles across its phenotypic spectrum has been recently extended to the pattern of microglial and astrocytic activation, which also appears subtype-specific [6, 11]. In this regard, the presence of a higher microglial immunoreactivity in sCJD VV2 compared to MM(V)1 subtype [11] consistently matched our findings of higher CSF levels of CHIT1 in the former compared to the latter. Consistently, but at variance with Llorens et al. who found similar YKL-40 levels in VV2 and MM(V)1 in both brain tissue and CSF [19], we also found that YKL-40 levels are particularly increased in VV2 cases, in line with those of CHIT1 and GFAP. Taken together, these findings indicate that the sCJD subtypes linked to the V2 prion strain, and especially the VV2, are characterized by a higher neuroinflammatory response that is possibly related to a more pronounced and widespread PrP^Sc^ deposition [11].

The present study also investigated for the first time the evolution of CSF glial and neurodegeneration markers in the prion disease group according to the disease stage. All glial and neurodegeneration proteins were significantly increased close to disease onset with CHIT1 and YKL-40 positively correlating with the disease stage. Moreover, CHIT1 and NfL, and to a lesser extent YKL-40 and GFAP, were all elevated 2 years before onset in our pre-symptomatic case of GSS.

All these findings support the notion that both glial activation and neuroaxonal degeneration are early phenomena in prion disease pathogenesis with the former showing a progressive increase along with the disease progression as previously described [11, 51]. Similarly, there is growing evidence that glial and NfL markers could help to track the disease in the pre-clinical phase of AD [52–54], but not in that of the FTD-ALS spectrum [23, 55, 56].

In terms of the distribution of glial markers across the FTD/FTLD spectrum, the ALS-FTD group showed higher values of CHIT1 compared to other groups as previously described [22], but this finding has now been extended to YKL-40 in our population. Overall, the FTLD-TDP group showed higher levels of CHIT1 and NfL and lower levels of p-tau/t-tau ratio than the FTLD-TAU group, thus reinforcing our preliminary findings obtained in a single-center cohort [33]. Accordingly, there were strong inter-correlations between CHIT1, NfL, and p-tau/t-tau ratio values in the FTD group (Additional file 1: Supplementary text). Based on these results, we speculate that the differences in NfL and p-tau/t-tau values might not be influenced by the presence of ALS pathology, while the distribution of CHIT1 levels appears to be influenced by both the motor neuron degeneration [22] and the type of proteinopathy. Interestingly, our findings were also confirmed after stratification according to the *CHIT1* genotype. However, if probable cases were excluded from the analysis, only the differences concerning NfL and p-tau/t-tau ratio were maintained between the TDP and TAU groups. In this regard, the less powerful difference in CHIT1 levels might also depend on the small size of the definite TAU group. Taken together, these findings suggest that neuroinflammation is a common pathophysiological mechanism in FTLD, the extent of which, however, may vary according to the distinct pathological phenotypes. For example, the higher levels of CHIT1 and YKL-40 but not of GFAP in TDP with ALS may indicate a higher expression/activation of a specific type of microgliosis/astrogliosis in the pyramidal tract and especially in the spinal cord.

The several inter-correlations we found between glial markers themselves and between glial and neurodegenerative markers in the three NDs (Additional file 1: Supplementary text) likely reflect the close relation between neuroinflammation and neurodegeneration, as previously described [15, 23, 54]. The same concept applies to the association between the biomarkers of amyloid-β and NFT accumulation with both YKL-40 and CHIT1 in our AD patients [15, 54]. At variance, the lack of correlation between YKL-40 and GFAP levels in both AD and FTD [23, 50] suggests that the two markers may reflect different astrocytic subpopulations or their distinct spatial distribution [23].

In terms of diagnostic value, we confirmed that only YKL-40 demonstrates a moderate accuracy with ≥ 80% sensitivity and specificity in discriminating between controls and AD or prion disease [19, 57], while neither YKL-40 nor CHIT1 or GFAP has a significant diagnostic value in any other comparison [18, 20, 57]. Finally, our results showed that NfL and p-tau/t-tau have a good diagnostic value in the discrimination between FTLD-TDP and FTLD-TAU [33, 48], while CHIT1 is less accurate.

The major strength of our study relates to the completeness and comprehensive characterization of the case series analyzed which comprise virtually all subtypes of both prion disease and FTD/FTLD spectrum, including several cases with a definite (pathological and/or genetic) diagnosis. On the other hand, the low proportion of autopsy-confirmed AD and FTLD-TAU subjects represents the main limitation. However, in each AD case, the in vivo diagnosis of AD was strongly supported by the positive CSF AD core biomarker profile. Similarly, we used AD core biomarkers to exclude AD co-pathology in each FTD case, and for each FTLD-TAU, the clinical diagnosis was supported by neuroimaging and follow-up data. Moreover, the fact that both centers participate in the Alzheimer’s Association quality control program on CSF biomarkers and that we did not find any significant differences in biomarker values across homogenous groups examined by the two centers speak against a significant inter-laboratory variability effect on our data [41, 58]. Given our choice to focus on distinct and “pure” proteinopathies associated with dementia, we did not purposely include dementia with Lewy bodies cases due to the large overlap with AD pathology. Moreover, our analysis of the evolution of CSF markers across disease stages in prion disease is partially speculative given its cross-sectional nature and the inclusion of a single preclinical case and needs to be confirmed in a larger, independent cohort. Finally, the fact that we could not obtain data regarding survival for AD and FTD patients may be considered an additional limit.

## Conclusions

Our results demonstrate a significant and largely overlapping increase in the levels of CHIT1, YKL-40, and GFAP in prion disease, AD, and FTLD, thus supporting the idea of a shared CSF neuroinflammatory profile in neurodegenerative dementias. The glial markers also showed different patterns across the clinicopathological subtypes of prion disease and FTLD and, most interestingly, across disease stages in prion disease. Thus, despite their poor diagnostic value, these glial biomarkers may be useful to track the ongoing neuroinflammatory process and to monitor the effects of newly developed neuroimmunomodulatory drugs.

## Supplementary information

**Additional file 1:** **Table S1.** Classification of prion disease (definite and probable) cases. **Table S2.** AD core biomarker values in controls, AD and FTD groups. **Table S3.** Multivariate linear regression models for CSF biomarker comparisons among diagnostic groups. **Figure S1.** Glial marker levels in distinct sCJD genotypes. **Table S4.** CSF biomarkers of neurodegeneration in prion disease subtypes. **Figure S2.** CSF biomarkers of neurodegeneration in sCJD molecular subtypes and genotypes. **Table S5.** Multivariate linear regression models for CSF biomarker comparisons among sCJD strains and molecular subtypes. **Table S6.** Distribution of CHIT1 levels in FTD proteinopathies according to CHIT1 genotype. **Table S7.** CSF biomarkers of neurodegeneration in the FTD/FTLD spectrum. **Figure S3.** CSF NfL and p-tau/t-tau in distinct FTD clinical syndromes and molecular subtypes. **Table S8.** Multivariate linear regression models for CSF biomarker comparisons among FTLD molecular subtypes. **Table S9.** CSF biomarkers of neuroinflammation and neurodegeneration in FTD mutation carriers. **Supplementary text.** CSF biomarkers within the FTD/FTLD spectrum after stratification according to the center. **Supplementary text.** CSF biomarkers inter-correlations.

## Data Availability

The datasets generated and analyzed during the present study are available from the corresponding author on reasonable request.

## Abbreviations

AD: Alzheimer’s disease
ALS-FTD: Amyotrophic lateral sclerosis associated with frontotemporal dementia
AUC: Area under the curve
Aβ: Beta-amyloid
bvFTD: Behavioral variant of frontotemporal dementia
c09orf72: Hexanucleotide repeat expansion on chromosome 9 open reading frame 72 gene
CBD: Corticobasal degeneration
CBS: Corticobasal syndrome
CHIT1: Chitotriosidase 1
CJD: Creutzfeldt-Jakob disease
CSF: Cerebrospinal fluid
CT: Computed tomography
CV: Coefficients of variation
DW: Diffusion weighted
ELISA: Enzyme-linked immunosorbent assay
FFI: Fatal familial insomnia
FLAIR: Fluid-attenuated inversion recovery
FTD: Frontotemporal dementia
FTLD: Frontotemporal lobar degeneration
FTLD-TAU: Frontotemporal lobar degeneration with tau pathology
FTLD-TDP: Frontotemporal lobar degeneration with TDP43 pathology
gCJD: Genetic Creutzfeldt-Jakob disease
gDNA: Genomic DNA
GFAP: Glial fibrillary acidic protein
GRN: Progranulin gene
GSS: Gerstmann-Sträussler-Scheinker syndrome
Het: Heterozygotes for CHIT1 24-bp duplication
Homo: Homozygotes for CHIT1 24-bp duplication
IQR: Interquartile range
LP: Lumbar puncture
M: Methionine
MAPT: Microtubule-associated protein tau gene
MMSE: Minimental State Examination
MM2C: MM2 cortical
MM2T: MM2 thalamic
MRI: Magnetic resonance imaging
MV2K: MV2 kuru type
NDs: Neurodegenerative dementias
NfL: Neurofilament light chain protein
nfvPPA: Nonfluent/agrammatic variant of primary progressive aphasia
PPA: Primary progressive aphasia
PrP^Sc^: Prion protein scrapie
PSP: Progressive supranuclear palsy
p-tau: Phosphorylated tau protein
ROC: Receiver operating characteristic
RT-QuIC: Real-time quaking-induced conversion
sCJD: Sporadic Creutzfeldt-Jakob disease
SD: Standard deviation
svPPA: Semantic variant of primary progressive aphasia
TARDBP: TAR DNA-binding protein 43 gene
t-tau: Total tau protein
V: Valine
VPSPr: Variably protease-sensitive prionopathy
WT: Wild type for CHIT1 24-bp duplication
YKL-40: Chitinase-3-like protein 1
