# Supplementary material for: CSF biomarkers of neuroinflammation in distinct forms and subtypes of neurodegenerative dementia
Source: Alzheimers Res Ther. 2019 Dec 31;12:2. doi: 10.1186/s13195-019-0562-4 (PMC6937795; doi:10.1186/s13195-019-0562-4)
Supplement: Supplementary file 1 — Additional file 1: Table S1. Classification of prion disease (definite and probable) cases. Table S2. AD core biomarker values in controls, AD and FTD groups. Table S3. Multivariate linear regression models for CSF biomarker comparisons among diagnostic groups. Figure S1. Glial marker levels in distinct sCJD genotypes. Table S4. CSF biomarkers of neurodegeneration in prion disease subtypes. Figure S2. CSF biomarkers of neurodegeneration in sCJD molecular subtypes and genotypes. Table S5. Multivariate linear regression models for CSF biomarker comparisons among sCJD strains and molecular subtypes. Table S6. Distribution of CHIT1 levels in FTD proteinopathies according to CHIT1 genotype. Table S7. CSF biomarkers of neurodegeneration in the FTD/FTLD spectrum. Figure S3. CSF NfL and p-tau/t-tau in distinct FTD clinical syndromes and molecular subtypes. Table S8. Multivariate linear regression models for CSF biomarker comparisons among FTLD molecular subtypes. Table S9. CSF biomarkers of neuroinflammation and neurodegeneration in FTD mutation carriers. Supplementary text. CSF biomarkers within the FTD/FTLD spectrum after stratification according to the center. Supplementary text. CSF biomarkers inter-correlations. [file 13195_2019_562_MOESM1_ESM.docx]

**ADDITIONAL FILE 1**

**Table S1. Classification of prion disease (definite and probable) cases.**

| **Subtype** | **N**  **Total** | **N Definite** | **Disease duration**  **(months)** | **N**  **Probable** | **Disease**  **Duration**  **(months)** | **RT-QuIC**  **Positive/**  **examined** | **Brain MRI**  **Positive/**  **examined** |
| --- | --- | --- | --- | --- | --- | --- | --- |
| **sCJD MM(V)1** | 34 | 34 | 3.53±2.82 | - | - | - | - |
| **sCJD VV2** | 26 | 18 | 6.28±2.27 | 8 | 6.08±4.36 | 8/8 | 5/7 |
| **sCJD MV2K** | 20 | 9 | 20.10±18.15 | 11 | 19.67±9.65 | 11/11 | 10/10 |
| **sCJD MM2C** | 4 | 2 | 40.5±6.36 | 2 | 34.5±17.8 | 2/2 | 2/2 |
| **sCJD VV1** | 1 | 1 | 13.5 | - | - | - | - |
| **VPsPr** | 1 | 1 | 34 | - | - | - | - |
| **gCJD E200K** | 5 | 5 | 2.73±1.05 | - | - | - | - |
| **gCJD V210I** | 5 | 5 | 4.37±4.01 | - | - | - | - |
| **FFI (D178N)** | 3 | 3 | 8.5 | - | - | - | - |
| **GSS (P102L)** | 2 | 2 | - | - | - | - | - |

*CJD* Creutzfeldt-Jakob disease*, FFI* fatal familial insomnia, *gCJD* genetic Creutzfeldt-Jakob disease, *GSS* Gerstmann-Sträussler-Scheinker syndrome, *MM(V)1* methionine homozygosity (valine) and scrapie prion protein type 1, *MM2C* methionine homozygosity and scrapie prion protein type 2, cortical type *MM2T* methionine homozygosity and scrapie prion protein type 2, thalamic type, *MRI* magnetic resonance imaging, *MV2K* methionine/valine heterozygosity and scrapie prion protein type 2, kuru type, *N* number, *RT-QuIC* real time quaking-induced conversion assay, *sCJD* sporadic Creutzfeldt-Jakob disease, *VPSPr* variably protease-sensitive prionopathy, *VV1* valine homozygosity and scrapie prion protein type 1, *VV2* valine homozygosity and scrapie prion protein type 2

**Table S2. AD core biomarker values in controls, AD and FTD groups.**

|  | **Controls** | **AD** | **FTD** |
| --- | --- | --- | --- |
| **N** | 40 | 40 | 72 |
| **t-tau** | 168 (138-228) | 698 (491-1013) | 253 (179-347) |
| **p-tau** | 39 (28-44) | 91 (72-118) | 36 (29-45) |
| **Aβ42** | 919 (643-1060) | 408 (321-480) | 820 (639-1011) |
| **Aβ40** | 8413 (5914-10400) | 10250 (6742-11750) | - |
| **t-tau/Aβ42** | 0.193 (0.152-0.287) | 1.803 (1.314-2.218) | 0.334 (0.231-0.439) |
| **p-tau/Aβ42** | 0.042 (0.032-0.052) | 0.214 (0.185-0.341) | 0.044 (0.036-0.056) |
| **Aβ42/Aβ40** | 1.066 (0.840-1.400) | 0.423 (0.352-0.512) | - |

*Aβ40* beta-amyloid 40, *Aβ42* beta-amyloid 42, *AD* Alzheimer’s disease, *FTD* frontotemporal dementia*, IQR* interquartile range, *N* number, *NfL* neurofilament light chain protein, *p-tau* phosphorylated tau protein, *t-tau* total tau protein

**Table S3. Multivariate linear regression models for CSF biomarker comparisons among diagnostic groups.**

|  | **Crude Coef (95% CI)** | **p** | **Adjusted Coef (95% CI)*** | **p** |
| --- | --- | --- | --- | --- |
| **CHIT1** | | | | |
| **Prion disease**  **AD**  **FTD** | 1.015 (0.597-1.435)  0.587 (0.085-1.088)  0.643 (0.201-1.085) | <0.001  0.022  0.005 | 1.014 (0.592-1.436)  0.590 (0.082-1.097)  0.636 (0.191-1.081) | <0.001  0.023  0.005 |
| **YKL-40** | | | | |
| **Prion disease**  **AD**  **FTD** | 0.892 (0.714-1.070)  0.549 (0.336-0.763)  0.353 (0.164-0.542) | <0.001  <0.001  <0.001 | 0.856 (0.682-1.029)  0.497 (0.181-0.548)  0.364 (0.288-0.705) | <0.001  <0.001  <0.001 |
| **GFAP** | | | | |
| **Prion disease**  **AD**  **FTD** | 0.588 (0.287-0.888)  0.515 (0.155-0.874)  0.561 (0.243-0.880) | <0.001  0.005  0.001 | 0.547 (0.253-0.841)  0.427 (0.075-0.780)  0.613 (0.302-0.925) | <0.001  0.018  0.001 |
| **t-tau** | | | | |
| **Prion disease**  **AD**  **FTD** | 3.174 (2.876-3.471)  1.358 (0.999-1.711)  0.134 (-0.089-0.431) | <0.001  <0.001  0.184 | 3.129 (2.835-3.423)  1.299 (0.946-1.651)  0.167 (-0.136-0.478) | <0.001  <0.001  0.195 |
| **NfL** | | | | |
| **Prion disease**  **AD**  **FTD** | 2.424 (2.149-2.670)  0.792 (0.463-1.121)  1.534 (1.244-1.824) | <0.001  <0.001  <0.001 | 2.410 (2.134-2.687)  0.767 (0.435-1.099)  1.543 (1.252-1.835) | <0.001  <0.001  <0.001 |

*AD* Alzheimer’s disease, *CHIT1* chitotriosidase 1*, CI* confidence interval, *CJD* Creutzfeldt-Jakob disease*, Coef* coefficient, *FTD* frontotemporal dementia, *GFAP* glial fibrillary acidic protein, *NfL* neurofilament light chain protein, *t-tau* total tau protein*, YKL-40* chitinase-3-like protein 1

y dependent variable: biomarker; x indipendent variables: diagnostic group (control group is the reference category), age, sex

*adjusted for age and sex

**Figure S1. Glial marker levels in distinct sCJD genotypes.** (A) CSF CHIT1 (all cases), (B) CSF CHIT1 (after the exclusion of the homozygotes for the 24bp duplication), (C) YKL-40 and (D) GFAP in sCJD MM, VV and MV genotypes. Horizontal lines represent medians. CHIT1 and GFAP values are expressed in logarithmic scale. Only statistically significant differences are displayed (Kruskal-Wallis followed by Dunn-Bonferroni post hoc test).


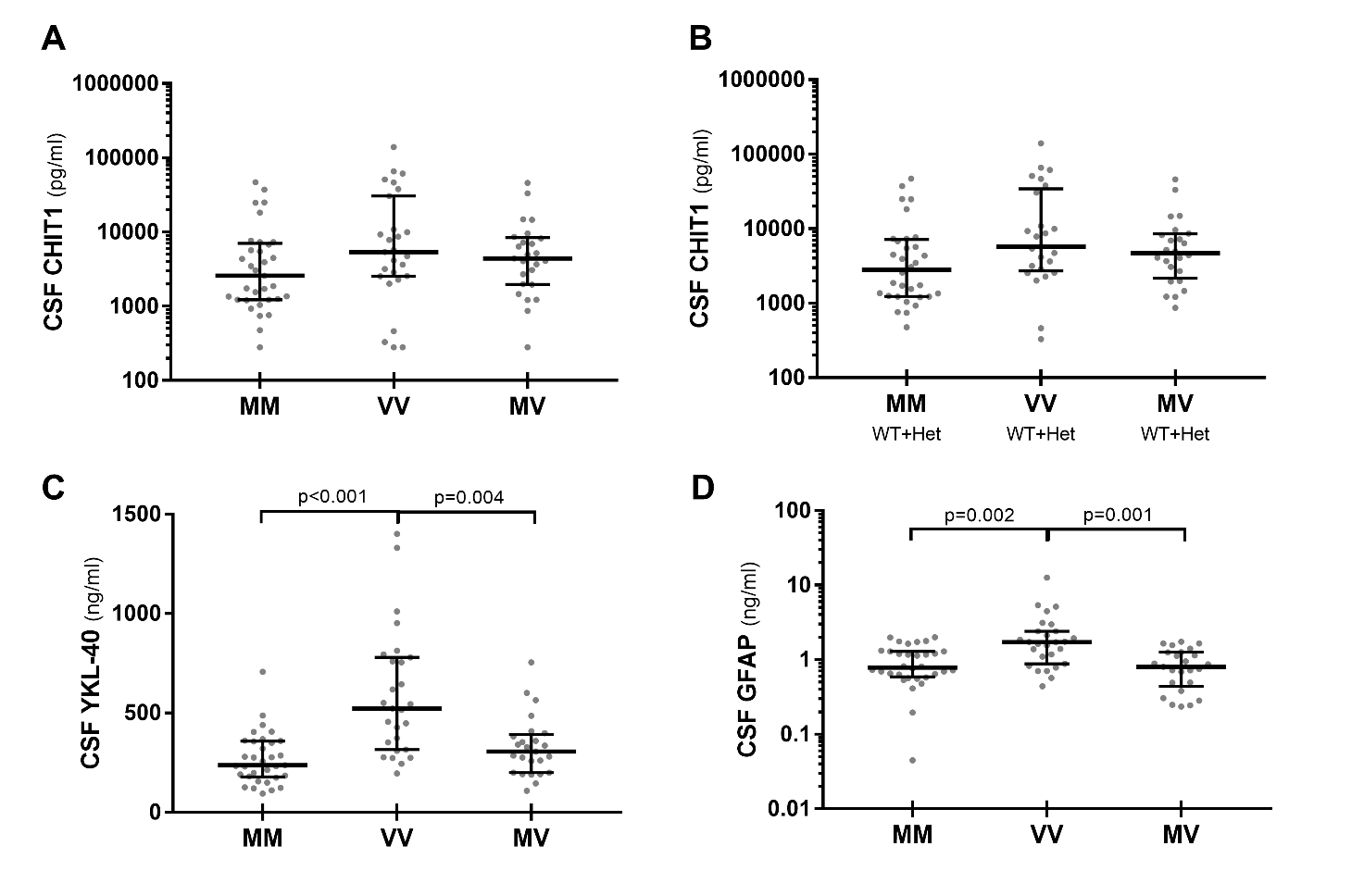


**Table S4. CSF biomarkers of neurodegeneration in prion disease subtypes.**

| **Subtype** | **N** | **t-tau**  **(pg/ml)**  **Median (IQR)** | **NfL**  **(pg/ml)**  **Median (IQR)** |
| --- | --- | --- | --- |
| **All sCJD** | 85 | 4825  (2352-10036) | 7875  (4244-12288) |
| ***sCJD MM(V)1*** | 34 | 7046  (2964-9480) | 5350  (3693-7100) |
| ***sCJD VV2*** | 26 | 10036  (4957-14825) | 12525  (10750-18150) |
| ***sCJD MV2K*** | 20 | 2032  (1455-3027) | 8900  (3700-12050) |
| ***sCJD MM2C*** | 4 | 1293  (950-3924) | 4294  (1785-8825) |
| ***sCJD VV1*** | 1 | 3790 | 15900 |
| **VPSPr** | 1 | 1273 | 1606 |
| **gCJD E200K** | 5 | 1881  (922-5703) | 6250  (3160-12775) |
| **gCJD V210I** | 5 | 6735  (5215-12850) | 5250  (3112-12300) |
| **FFI (D178N)** | 3 | 288, 120, 190 | 5150, 1881, 3536 |
| **Presymptomatic**  **GSS (P102L)** | 1 | 459* | 8146* |
| **Symptomatic GSS (P102L)** | 2 | 1969*, 566 | 20690*, 2611 |

*CJD* Creutzfeldt-Jakob disease*, FFI* fatal familial insomnia, *gCJD* genetic Creutzfeldt-Jakob disease, *GSS* Gerstmann-Sträussler-Scheinker syndrome, *IQR* interquartile range, *MM(V)1* methionine homozygosity (valine) and scrapie prion protein type 1, *MM2C* methionine homozygosity and scrapie prion protein type 2, cortical type *MM2T* methionine homozygosity and scrapie prion protein type 2, thalamic type, *MV2K* methionine/valine heterozygosity and scrapie prion protein type 2, kuru type, *N* number, *NfL* neurofilament light protein, *sCJD* sporadic Creutzfeldt-Jakob disease, *VPSPr* variably protease-sensitive prionopathy, *VV1* valine homozygosity and scrapie prion protein type 1, *VV2* valine homozygosity and scrapie prion protein type 2, *t-tau* total tau protein

* same patient

**Figure S2. CSF biomarkers of neurodegeneration in sCJD molecular subtypes and genotypes.** (A) CSF t-tau in sCJD MM(V)1, VV2 and MV2K subtypes; (B) CSF t-tau in CJD MM, VV and MV genotypes, (C) NfL in sCJD MM(V)1, VV2 and MV2K subtypes; (D) NfL in CJD MM, VV and MV genotypes. Horizontal lines represent medians. T-tau and NfL values are expressed in logarithmic scale. Only statistically significant differences are displayed (Kruskal-Wallis followed by Dunn-Bonferroni post hoc test).

**
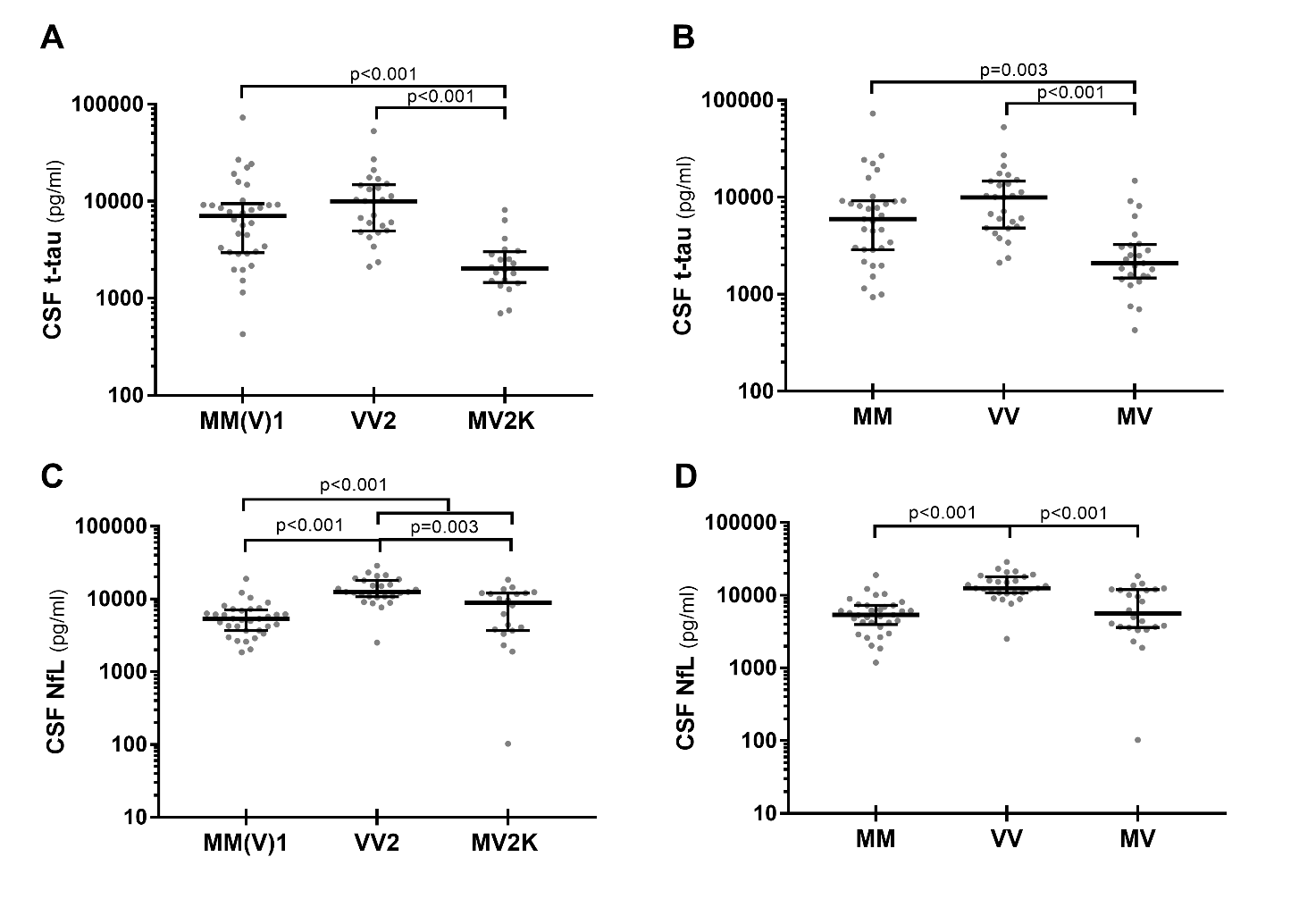
**

**Table S5. Multivariate linear regression models for CSF biomarker comparisons among sCJD strains and molecular subtypes.**

|  | **Crude Coef (95% CI)** | **P** | **Adjusted Coef (95% CI)*** | **p** |  |
| --- | --- | --- | --- | --- | --- |
| **CHIT1**** | | | | |  |
| **Strain V2 vs M1**  **VV2 vs MM(V)1**  **MV2K vs MM(V)1** | 0.642 (0.065-1.219)  0.857 (0.184-1.530)  0.395 (-0.307-1.097) | 0.030  0.013  0.265 | 0.640 (0.052-1.228)  0.860 (0.177-1.542)  0.365 (-0.367-1.097) | 0.033  0.014  0.323 |  |
| **YKL-40** | | | | |  |
| **Strain V2 vs M1**  **VV2 vs MM(V)1**  **MV2K vs MM(V)1** | 0.533 (0.297- 0.769)  0.752 (0.497-1.007)  0.248 (-0.028-0.524) | <0.001  <0.001  0.077 | 0.543 (0.321- 0.765)  0.723 (0.478-0.968)  0.296 (0.026-0.567) | <0.001  <0.001  0.052 |  |
| **GFAP** | | | | |  |
| **Strain V2 vs M1**  **VV2 vs MM(V)1**  **MV2K vs MM(V)1** | 0.359 (-0.009- 0.726)  0.768 (0.387-1.149)  -0.173 (-0.584-0.238) | 0.066  <0.001  0.404 | 0.377 (-0.017- 0.737)  0.740 (0.357-1.124)  -0.117 (-0.538-0.305) | 0.054  <0.001  0.583 |  |
| **t-tau** | | | | |  |
| **Strain V2 vs M1**  **VV2 vs MM(V)1**  **MV2K vs MM(V)1** | -0.235 (-0.688-0.217)  0.383 (-0.059-0.824)  -1.039 (-1.517- -0.561) | 0.303  0.089  <0.001 | -0.239 (-0.685-0.206)  0.382 (-0.050-0.813)  -1.093 (-1.570- -0.616) | 0.288  0.082  <0.001 |  |
| **NfL** | | | | |  |
| **Strain V2 vs M1**  **VV2 vs MM(V)1**  **MV2K vs MM(V)1** | 0.565 (0.213-0.918)  0.913 (0.538-1.287)  0.114 (-0.291- -0.518) | 0.002  <0.001  0.578 | 0.568 (0.215-0.920)  0.903 (0.523-1.283)  0.118 (-0.299- -0.534) | 0.002  <0.001  0.576 |  |

*CHIT1* chitotriosidase 1*, CI* confidence interval, *CJD* Creutzfeldt-Jakob disease*, Coef* coefficient, *GFAP* glial fibrillary acidic protein, *MM(V)1* methionine homozygosity (valine) and scrapie prion protein type 1, *MV2K* methionine/valine heterozygosity and scrapie prion protein type 2, kuru type, *N* number, *NfL* neurofilament light chain protein, *t-tau* total tau protein*, VV2* valine homozygosity and scrapie prion protein type 2, *YKL-40* chitinase-3-like protein 1,

y dependent variable: biomarker; x indipendent variables: diagnostic group, age, sex

*adjusted for age and sex; ** after the exclusion of the homozygotes for the 24bp duplication

**Table S6.** **Distribution of CHIT1 levels in FTD proteinopathies according to CHIT1 genotype.**

|  |  | **CHIT1 levels**  **Median (IQR)**  **pg/ml** | | |
| --- | --- | --- | --- | --- |
|  | **N** | **WT^a^** | **Het^b^** | **Homo** |
| **FTLD-TDP** | 34 | 5886 (3120-13213)  [N=23] | 1894 (1034-5743)  [N=8] | 280  [N=1] |
| **FTLD-TAU** | 38 | 2466 (1666-4691)  [N=21] | 680 (541-1094)  [N=9] | 280  [N=2] |

*CHIT1* chitotriosidase 1, *FTLD-TAU* frontotemporal lobar degeneration with tau pathology, *FTLD-TDP* frontotemporal lobar degeneration with TDP43 pathology, *Het* heterozygotes for CHIT1 24bp duplication*, Homo* homozygotes for CHIT1 24bp duplication*, IQR* interquartile range, *N* number, *WT* wild type for CHIT1 24bp duplication

^a^ Kruskal-Wallis followed by Dunn-Bonferroni post hoc test: FTLD-TDP vs. FTLD-TAU p=0.007; FTLD-TDP with ALS vs. FTLD-TAU p=0.001; FTLD-TDP without ALS vs. TAU p=0.040; FTLD-TDP with ALS vs. FTLD-TDP without ALS p=0.032

^b^ Mann Whitney *U* test: FTLD-TDP vs. FTLD-TAU p=0.012

**Table S7.** **CSF biomarkers of neurodegeneration in the FTD/FTLD spectrum.**

|  | **N** | **NfL^a^**  **(pg/ml)**  **Median (IQR)** | **p-tau/t-tau^b^**  **Median (IQR)** |
| --- | --- | --- | --- |
| **Clinical diagnosis** | | | |
| **bvFTD** | 17 | 4226 (1879-5700) | 0.137 (0.085-0.154) |
| **PPA** | 8 | 4346 (3478-9943) | 0.106 (0.082-0.138) |
| ***nfvPPA*** | 6 | 4286  (3086-17384) | 0.106 (0.084-0.170) |
| ***svPPA*** | 2 | 3848, 7642 | 0.117, 0.078 |
| **ALS-FTD** | 9 | 7000 (4590-10600) | 0.115 (0.067-0.184) |
| **PSP** | 23 | 1440 (1153-2783) | 0.193 (0.134-0.221) |
| **CBS** | 12 | 1464 (1257-2924) | 0.164 (0.144-0.182) |
| **FTD+ parkinsonism** | 3 | 1865; 1970, 8150 | 0.196, 0.107, 0.101 |
| **Proteinopathies** | | | |
| **FTLD-TAU** | 38 | 1465 (1213-2827) | 0.182 (0.142-0.214) |
| **FTLD-TDP** | 34 | 4834 (3000-7769) | 0.117 (0.085-0.149) |
| ***TDP without ALS***  ***TDP with ALS*** | 25  9 | 4305 (2145-6946)  7000 (4590-10600) | 0.117 (0.085-0.149)  0.115 (0.067-0.184) |

*ALS* amyotrophic lateral sclerosis, *ALS-FTD* amyotrophic lateral sclerosis associated with frontotemporal dementia, *bvFTD* behavioural variant of frontotemporal dementia, *CBS* corticobasal syndrome, *FTD* frontotemporal dementia, *FTLD-TAU* frontotemporal lobar degeneration with tau pathology, *FTLD-TDP* frontotemporal lobar degeneration with TDP43 pathology, *N* number, *NfL* neurofilament light chain protein, *nfvPPA* nonfluent/agrammatic variant of primary progressive aphasia, *PPA* primary progressive aphasia, *PSP* progressive supranuclear palsy, *p-tau* phosphorylated tau protein, *svPPA* semantic variant of primary progressive aphasia, *t-tau* total tau protein

^a^ Mann Whitney *U* test: definite FTLD-TDP vs. definite FTLD-TAU p=0.046

^b^ Mann Whitney *U* test: definite FTLD-TDP vs. definite FTLD-TAU p=0.016

**Figure S3. CSF NfL and p-tau/t-tau in distinct FTD clinical syndromes and molecular subtypes.** (A) CSF NfL in FTD clinical groups; (B) CSF NfL in FTLD-TAU, FTLD-TDP without ALS and FTLD-TDP with ALS; (C) CSF p-tau/t-tau in FTD clinical groups; (D) CSF p-tau/t-tau in FTLD-TAU, FTLD-TDP without ALS and FTLD-TDP with ALS. Horizontal lines represent medians. NfL ad p-tau/t-tau values are expressed in logarithmic scale. Only statistically significant differences are displayed (Kruskal-Wallis followed by Dunn-Bonferroni post hoc test).

**
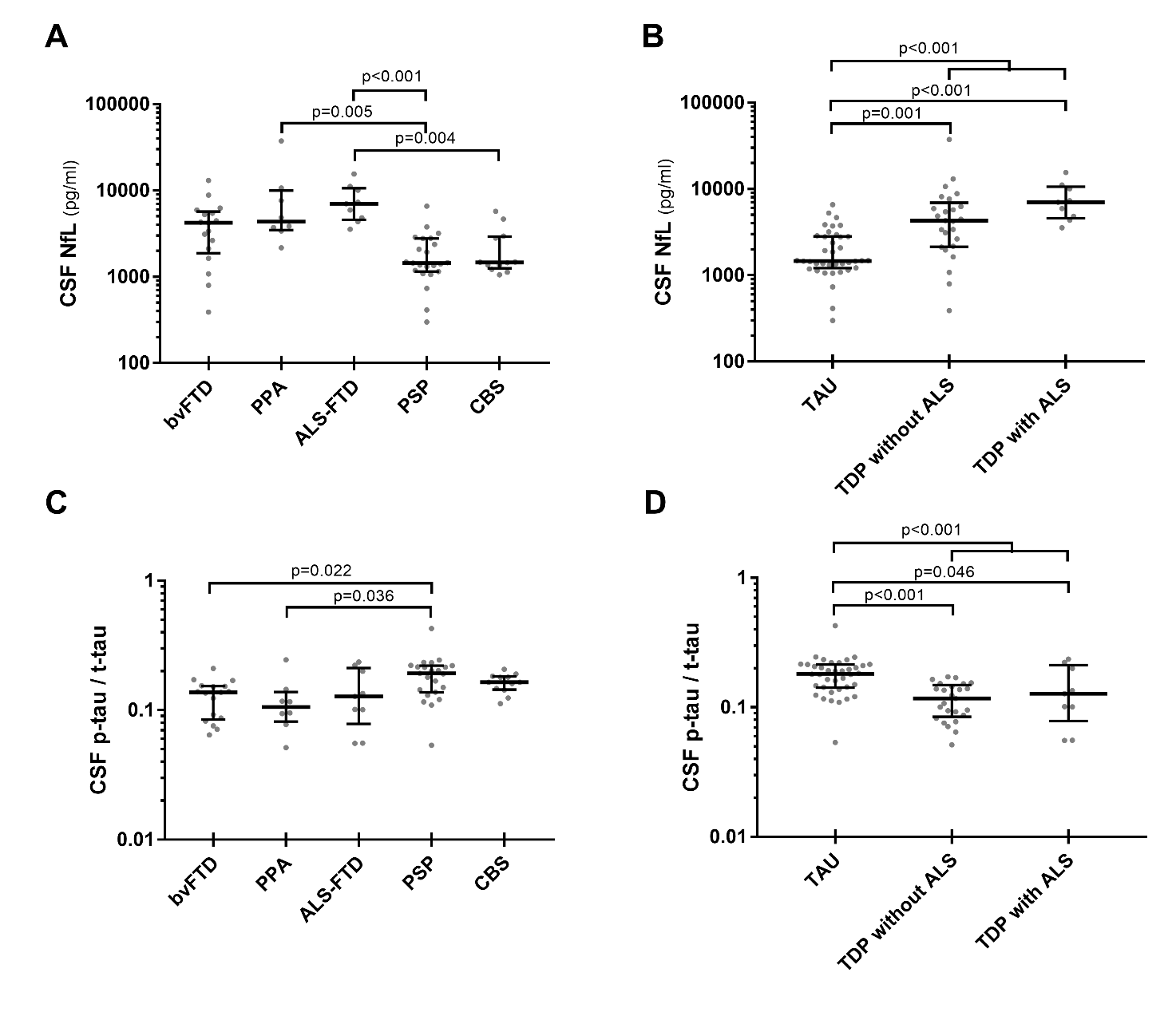
**

**Table S8. Multivariate linear regression models for CSF biomarker comparisons among FTLD molecular subtypes.**

|  | **Crude Coef (95% CI)** | **P** | **Adjusted Coef (95% CI)*** | **P** |
| --- | --- | --- | --- | --- |
| **CHIT1** | | | | |
| **FTLD-TAU vs FTLD TDP** | -0.890 (-1.372- -0.407) | <0.001 | -0.845 (-1.372- -0.319) | <0.001 |
| **YKL-40** | | | | |
| **FTLD-TAU vs FTLD TDP** | -0.006 (-0.237-0.225) | 0.957 | -0.079 (-0.328-0.171) | 0.530 |
| **GFAP** | | | | |
| **FTLD-TAU vs FTLD TDP** | -0.248 (-0.642-0.147) | 0.214 | -0.350 (-0.763-0.063) | 0.214 |
| **NfL** | | | | |
| **FTLD-TAU vs. FTLD TDP** | -0.965 (-1.326- -0.605) | <0.001 | -0.939 (-1.333- -0.545) | <0.001 |
| **p-tau/t-tau** | | | | |
| **FTLD-TAU vs. FTLD TDP** | 0.444 (0.277-0.612) | <0.001 | 0.473 (0.293-0.652) | <0.001 |

*CHIT1* chitotriosidase 1*, CI* confidence interval, *Coef* coefficient, *FTLD-TAU* frontotemporal lobar degeneration with tau pathology, *FTLD-TDP* frontotemporal lobar degeneration with TDP43 pathology, *GFAP* glial fibrillary acidic protein, *NfL* neurofilament light chain protein, *p-tau* phosphorylated tau protein, *t-tau* total tau protein*, YKL-40* chitinase-3-like protein 1

y dependent variable: biomarker; x indipendent variables: diagnostic group, age, sex

*adjusted for age and sex;

**Table S9. CSF biomarkers of neuroinflammation and neurodegeneration in FTD mutation carriers.**

|  | **N** | **CHIT1**  **All**  **(pg/ml)**  **Median (IQR)** | **CHIT1**  **WT+Het**  **(pg/ml)**  **Median (IQR)** | **YKL-40**  **(pg/ml)**  **Median (IQR)** | **GFAP**  **(pg/ml)**  **Median (IQR)** | **NfL**  **(pg/ml)**  **Median (IQR)** | **p-tau/t-tau^a^**  **Median (IQR)** |
| --- | --- | --- | --- | --- | --- | --- | --- |
| ***C9orf72*** | 19 | 4575 (1966-14900) | 5947  (2543-15104) | 184  (134-255) | 1.248  (0.725-1.594) | 5749  (3119-8150) | 0.134  (0.101-0.152) |
| ***GRN*** | 12 | 3676 (2069-7098) | 3676 (2069-7098) | 252  (162-268) | 1.223  (0.630-1.802) | 4947  (2436-8548) | 0.093  (0.076-0.134) |
| ***TARDBP*** | 2 | 11500, 1664 | 11500, 1664 | 290, 121 | 0.312, 1.226 | 3565, 2166 | 0.223, 0.095 |
| ***MAPT*** | 4 | 1705  (1249-4599) | 1705  (1249-4599) | 190  (168-288) | 1.190 (0.900-1.524) | 3791  (2332-4909) | 0.203  (0.137-0.236) |

*CHIT1* chitotriosidase 1*, c09orf72* hexanucleotide repeat expansion on chromosome 9 open reading frame 72 gene, *CHIT1* chitotriosidase 1*, GFAP* glial fibrillary acidic protein, *GRN* progranulin gene, *Het* heterozygotes for CHIT1 24bp duplication*, IQR* interquartile range, *MAPT* microtubule-associated protein tau gene, *N* number, *NfL* neurofilament light chain protein, *p-tau* phosphorylated tau protein, *TARDBP* TAR DNA-binding protein 43 gene, *t-tau* total tau protein*, WT* wild type for CHIT1 24bp duplication, *YKL-40* chitinase-3-like protein 1

^a^ *GRN* carriers vs. *MAPT* carriers p=0.039

**Supplementary text. CSF biomarkers within the FTD/FTLD spectrum after stratification according to the center.**

No differences in CHIT1 (p=0.133), YKL-40 (p=0.536), GFAP (p=0.887), t-tau (p=0.417), NfL (p=0.134), p-tau (p=0.070) and Aβ42 (p=0.230) levels were detected between bvFTD cases from Bologna (n=10) and those from Ulm (n=7). No differences in CHIT1 (p=0.067), YKL-40 (p=0.346), GFAP (p=0.872), t-tau (p=0.343), NfL (p=0.077), p-tau (p=0.131) and Aβ42 (p=0.832) levels were detected between PSP cases from Bologna (n=10) and those from Ulm (n=13). No differences in CHIT1 (p=0.084), YKL-40 (p=1.000), GFAP (p=0.242), t-tau (p=0.059), NfL (p=0.889), p-tau (p=0.195) and Aβ42 (p=0.197) levels were detected between FTLD-TDP cases from Bologna (n=21) and those from Ulm (n=13). No differences in CHIT1 (p=0.222), YKL-40 (p=0.802), GFAP (p=0.802), t-tau (p=0.159), NfL (p=0.461), p-tau (p=0.558) and Aβ42 (p=0.189) levels were detected between FTLD-TAU cases from Bologna (n=20) and those from Ulm (n=18).

After exclusion of the FTD Ulm cohort (n=31), the FTD group showed higher levels of CHIT1 (p=0.026), YKL-40 (p=0.003), GFAP (p=0.010), and NfL (p<0.001) compared to controls; lower levels of t-tau (p<0.001), NfL (p<0.001) and YKL-40 (p<0.001) compared to CJD, but similar levels of CHIT1 and GFAP to those of AD and CJD. Among FTD clinical syndromes, CSF CHIT1 and NfL levels were still higher in ALS-FTD than in CBS (p=0.014, p=0.021 respectively) or PSP (p=0.002, p=0.003 respectively). We did not confirm the significantly higher levels of YKL-40 in FTD-ALS compared to CBS and bvFTD possibly because of the smaller sample size. FTLD-TDP cases showed higher CHIT1 levels (p<0.001) and NfL (p<0.001) but lower values of p-tau/t-tau ratio (p<0.001) than those with FTLD-TAU. The analysis demonstrated increased CHIT1 and NfL levels in comparison to TAU not only in TDP with ALS (p<0.001, p<0.001 respectively) but also in TDP without ALS (p=0.016, p=0.005 respectively), but no difference in CHIT1 and NfL between TDP with ALS and those without ALS. On the contrary p-tau/t-tau ratio values were higher in TAU compared to both TDP with ALS (p<0.001) and TDP without ALS (p=0.016). TDP with ALS showed higher levels of YKL-40 compared to TDP without ALS (p=0.027) and TAU (p=0.026). Finally, GFAP and t-tau showed comparable values among FTD clinical syndromes and proteinopathies.

**Supplementary text. CSF biomarkers inter-correlations.**

In prion disease patients, CHIT1 moderately correlated with YKL-40 (Spearman’s rho =0.319, p=0.001) and NfL (Spearman’s rho =0.350, p<0.001); while YKL-40 was associated with t-tau (Spearman’s rho =0.347, p<0.001) NfL (Spearman’s rho =0.503, p<0.001) and GFAP (Spearman’s rho=0.333, p=0.001). There were also correlations between GFAP and t-tau (Spearman’s rho=0.285, p=0.004) or NfL (Spearman’s rho =0.325, p=0.001). In the same group, t-tau levels correlated with NfL (Spearman’s rho =0.400, p < 0.001).

In AD there were moderate associations between CHIT1 levels and YKL-40 (r =0.386, p =0.014), NfL (Spearman’s rho =0.380, p=0.016), t-tau (Spearman’s rho =0.426, p=0.006), p-tau (Spearman’s rho=0.493, p=0.001), Aβ42/Aβ40 ratio (Spearman’s rho =-0.349, p=0.032) and Aβ40 values (Spearman’s rho =0.521, p=0.001). Moreover, YKL-40 correlated with t-tau (Spearman’s rho =0.382, p=0.015), p-tau (Spearman’s rho =0.426, p=0.006) and Aβ42/ Aβ40 ratio (Spearman’s rho =-0.431, p=0.007). Otherwise, GFAP was associated only with NfL (Spearman’s rho =0.364, p=0.021). NfL, t-tau and p-tau were also inter-correlated.

Finally, in FTD cases CHIT1 correlated with YKL-40 (Spearman’s rho =0.264, p=0.026), t-tau (Spearman’s rho =0.339, p=0.004), NfL (Spearman’s rho =0.564, p<0.001) and p-tau/t-tau ratio (Spearman’s rho =-0.276, p=0.019), which were inter-correlated (Spearman’s rho =-0.533, p<0.001), whereas YKL-40 was associated with NfL (Spearman’s rho =0.465, p<0.001) and t-tau (Spearman’s rho =0.402, p=0.001). GFAP slightly correlated with t-tau (Spearman’s rho =0.281, p=0.019) and p-tau/t-tau (Spearman’s rho =-0.268, p=0.025).
